# VEGF-induced intracellular Ca^2+^ oscillations are down-regulated and do not stimulate angiogenesis in breast cancer-derived endothelial colony forming cells

**DOI:** 10.18632/oncotarget.20255

**Published:** 2017-08-14

**Authors:** Francesco Lodola, Umberto Laforenza, Fabio Cattaneo, Federico Alessandro Ruffinatti, Valentina Poletto, Margherita Massa, Richard Tancredi, Estella Zuccolo, Dlzar Alì Khdar, Alberto Riccardi, Marco Biggiogera, Vittorio Rosti, Germano Guerra, Francesco Moccia

**Affiliations:** ^1^ Laboratory of General Physiology, Department of Biology and Biotechnology “Lazzaro Spallanzani”, University of Pavia, Pavia 27100, Italy; ^2^ Department of Molecular Medicine, University of Pavia, Pavia 27100, Italy; ^3^ Department of Molecular Medicine and Medical Biotechnology, University of Naples Federico II, Naples 80131, Italy; ^4^ Department of Life Sciences and Systems Biology, Turin 10123, Italy; ^5^ Laboratory of Biochemistry, Biotechnology and Advanced Diagnosis, Foundation IRCCS Policlinico San Matteo, Pavia 27100, Italy; ^6^ Laboratory of Immunology Transplantation, Foundation IRCCS Policlinico San Matteo, Pavia 27100, Italy; ^7^ Medical Oncology Unit, Foundation IRCCS Salvatore Maugeri, Pavia 27100, Italy; ^8^ Department of Internal Medicine, University of Pavia, Pavia 27100, Italy; ^9^ Laboratory of Cell Biology and Neurobiology, Department of Biology and Biotechnology “Lazzaro Spallanzani”, University of Pavia, Pavia 27100, Italy; ^10^ Department of Medicine and Health Sciences “Vincenzo Tiberio”, University of Molise, Campobasso 86100, Italy; ^11^ Current address: Italian Institute of Technology, Center for Nano Science and Technology, Milano 20133, Italy

**Keywords:** VEGF, breast cancer, endothelial colony forming cells, intracellular Ca^2+^ oscillations, angiogenesis

## Abstract

Endothelial colony forming cells (ECFCs) represent a population of truly endothelial precursors that promote the angiogenic switch in solid tumors, such as breast cancer (BC). The intracellular Ca^2+^ toolkit, which drives the pro-angiogenic response to VEGF, is remodelled in tumor-associated ECFCs such that they are seemingly insensitive to this growth factor. This feature could underlie the relative failure of anti-VEGF therapies in cancer patients. Herein, we investigated whether and how VEGF uses Ca^2+^ signalling to control angiogenesis in BC-derived ECFCs (BC-ECFCs). Although VEGFR-2 was normally expressed, VEGF failed to induce proliferation and *in vitro* tubulogenesis in BC-ECFCs. Likewise, VEGF did not trigger robust Ca^2+^ oscillations in these cells. Similar to normal cells, VEGF-induced intracellular Ca^2+^ oscillations were triggered by inositol-1,4,5-trisphosphate-dependent Ca^2+^ release from the endoplasmic reticulum (ER) and maintained by store-operated Ca^2+^ entry (SOCE). However, InsP_3_-dependent Ca^2+^ release was significantly lower in BC-ECFCs due to the down-regulation of ER Ca^2+^ levels, while there was no remarkable difference in the amplitude, pharmacological profile and molecular composition of SOCE. Thus, the attenuation of the pro-angiogenic Ca^2+^ response to VEGF was seemingly due to the reduction in ER Ca^2+^ concentration, which prevents VEGF from triggering robust intracellular Ca^2+^ oscillations. However, the pharmacological inhibition of SOCE prevented BC-ECFC proliferation and *in vitro* tubulogenesis. These findings demonstrate for the first time that BC-ECFCs are insensitive to VEGF, which might explain at cellular and molecular levels the failure of anti-VEGF therapies in BC patients, and hint at SOCE as a novel molecular target for this disease.

## INTRODUCTION

Breast cancer (BC) consists in a heterogeneous group of malignancies deriving from the epithelial cells lining the milk ducts and still represents the leading cause of cancer-related mortality in women worldwide [1]. BC growth and metastasis are supported by the development of an intricate network of blood vessels which nourish cancer cells with oxygen and nutrients and remove their catabolic waste [2]. An increase in microvascular density positively correlates with the degree of metastasis, tumor recurrence and poorer outcome of invasive BC [3], which further hints at neovascularisation as a key determinant of BC malignancy. Vascular endothelial growth factor (VEGF) comprises a family of secreted polypeptides which include VEGF-A, VEGF-B, VEGF-C, VEGF-D and placental growth factor (PLGF) and act through three cognate receptor tyrosine kinases, designated as VEGFR1 (Flt1), VEGFR2 (Flk1/KDR), and VEGFR3 (Flt4) [4]. VEGF-A (hereafter termed as VEGF) is the prototypical member of the family and constitutes a master regulator of vascular development in health and disease [4–6]. VEGFR2 serves as the main transducer of VEGF signalling on endothelial cell differentiation, proliferation, migration and tubulogenesis [4]. Consistently, targeting VEGFR2 with either humanized monoclonal antibodies, such as bevacizumab and VEGF-Trap, or tyrosine kinase inhibitors (TKIs), such as sorafenib, sunitinib and pazopanib, halted neovessel formation and caused tumor shrinkage in immunodeficient mouse models of most malignancies [7, 8], including BC. Regrettably, these promising observations did not translate into an effective therapeutic benefit for cancer patients. Clinical trials have shown that anti-VEGF drugs, administered either as monotherapy or in combination with chemotherapy or radiation therapy, merely slowed down tumor progression, significantly prolonging only progression free survival (PFS), without improving overall survival (OS) in these individuals [7, 8]. A recent meta-analysis of randomised phase II and III clinical trials of bevacizumab (Avastin) revealed a lack of any substantial OS benefit either in the neoadjuvant or in the metastatic setting in BC patients [9]. Moreover, in 2010, the Food and Drug Administration (FDA) removed approval of Avastin for metastatic BC because of its toxicity and disease progression during long-term treatment [10]. The efficacy of anti-angiogenic therapy is severely limited by the development of refractoriness to anti-VEGF drugs, which causes tumor relapse after an initial period of tumor shrinkage or stasis. Also, a minority of cancer patients are intrinsically resistant to angiogenesis blockade, such that disease progression continues unabated and leads to patient death [11]. The failure of anti-VEGF drugs has been attributed to several mechanisms, such as the recruitment of VEGF-insensitive endothelial progenitor cells (EPCs), which are mobilized from either the bone marrow or the arterial wall and support angiogenesis in several types of cancer [12, 13], including BC. Among the several EPC subsets that have described in peripheral blood, endothelial colony forming cells (ECFCs) represent the only truly endothelial precursors [14], anastomose with the host vasculature and form patent vessels *in vivo* [14, 15], display an innate tumor tropism [13, 16–18] and may therefore drive the angiogenic switch by supplying endothelial cells to growing neovessels in BC and many other types of tumors, including BC [19–21]. A recent study revealed that ECFC frequency is remarkably increased in peripheral blood of naïve, i.e. not treated, BC patients [22]. Interestingly, VEGF fails to stimulate proliferation and *in vitro* tubulogenesis in ECFCs isolated from subjects suffering from solid tumors [23], such as renal cell carcinoma (RCC) [24] and infantile hemangioma (IH) [25], as well as in primary myelofibrosis (PMF) [26]. The effect of VEGF on BC-associated ECFCs (BC-ECFCs) is, however, still unknown.

VEGF has recently been shown to stimulate ECFC proliferation by inducing repetitive oscillations in intracellular Ca^2+^ concentration ([Ca^2+^]_i_) [27–29], which in turn promote the nuclear translocation of the Ca^2+^-sensitive transcription factor, NF-κB. Upon binding to its agonist, VEGFR2 recruits phospholipase Cγ (PLCγ) to synthesize inositol-1,4,5-trisphosphate (InsP_3_), which triggers the rhythmical Ca^2+^ discharge from the endoplasmic reticulum (ER), the largest Ca^2+^ reservoir in ECFCs [30]. VEGF-induced Ca^2+^ oscillations are sustained over time by the so-called store-operated Ca^2+^ entry (SOCE) mechanism [28], which is initiated by the activation of the ER Ca^2+^ sensor Stim1 following InsP_3_-induced ER Ca^2+^ depletion [31]. Once activated, Stim1 translocates towards the most peripheral regions of ER, where it traps and gates the two ubiquitous store-operated Ca^2+^-permeable channels, Orai1 and Transient Receptor Channel Canonical 1 (TRPC1) [24, 31, 32]. The Ca^2+^ toolkit is severely dysregulated in tumor-associated ECFCs [23, 27, 33, 34]]. For instance, the ER Ca^2+^ content is significantly reduced in RCC- and IH-derived ECFCs (RCC-ECFCs and IH-ECFCs, respectively) [25, 35], which might prevent VEGF from eliciting the periodical Ca^2+^ release [23]. Conversely, SOCE is up-regulated and controls proliferation in both RCC-ECFCs [24] and IH-ECFCs [25], thereby standing out as an alternative, promising target for highly angiogenic tumors [33, 36]. Of note, preliminary results indicated that VEGF-induced pro-angiogenic Ca^2+^ oscillations could be attenuated also in BC-ECFCs [22].

The present investigation was endeavoured to assess whether and how VEGF stimulates pro-angiogenic Ca^2+^ oscillations in BC-ECFCs. We exploited a multi-disciplinary approach, comprising electron microscopy (EM), Ca^2+^ imaging, real-time polymerase chain reaction (qRT-PCR), Western blotting, and functional assays to demonstrate that VEGF fails to promote proliferation and *in vitro* tubulogenesis in BC-ECFCs due to the down-regulation of the underlying repetitive Ca^2+^ spikes. The suppression of the Ca^2+^-dependent response to VEGF involves the decrease in ER Ca^2+^ levels. Conversely, SOCE is still functional in these cells and can be targeted to inhibit BC-ECFC proliferation. Our data contribute to shed light at cellular and molecular level on the failure of anti-VEGF therapies and hint at SOCE as an alternative target to halt vascularization in this disease.

## RESULTS

### Ultrastructural analysis reveals that BC-ECFCs are morphologically different as compared to normal cells

**Figure 1:**
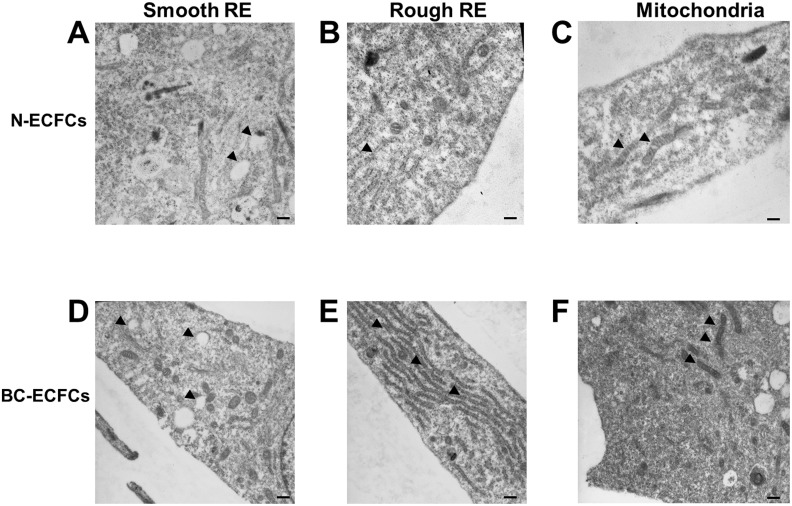
Ultrastructural remodelling in breast cancer-derived endothelial colony forming cells Ultrastructural analysis of endothelial colony forming cell morphology. Representative images of two N-ECFCs and two BC-ECFCs samples analyzed. The bars in **(A-F)** correspond to 500 nm.

A recent microarray analysis unveiled that the genomic profile of BC-ECFCs was dramatically altered as compared to normal cells (N-ECFCs) as indicated by the identification of 342 differentially expressed genes (DEGs; 192 up-regulated, 150 down-regulated) in the former [22]. In order to assess whether this remarkable difference in the gene signature was associated to a significant ultrastructural rearrangement, as recently shown for RCC-ECFCs [35], we carried out a throughout analysis at electron microscope levels. This investigation revealed clear ultrastructural differences between N- and BC-ECFCs. Figure 1A and Figure 1D show that smooth ER (sER) vesicles were more abundant in BC-ECFCs. Likewise, rough ER (rER) cisternae occupied a much larger area and were more closely packed in BC-ECFCs as compared to normal cells (Figure 1B and Figure 1E). Finally, mitochondria were also more numerous and enlarged in BC-ECFCs (Figure 1C and Figure 1F). Therefore, not only the gene signature profile [22], but also the cellular microarchitecture was dramatically remodeled in BC-ECFCs.

### VEGF does not stimulate proliferation and *in vitro* tubulogenesis in BC-ECFCs

**Figure 2:**
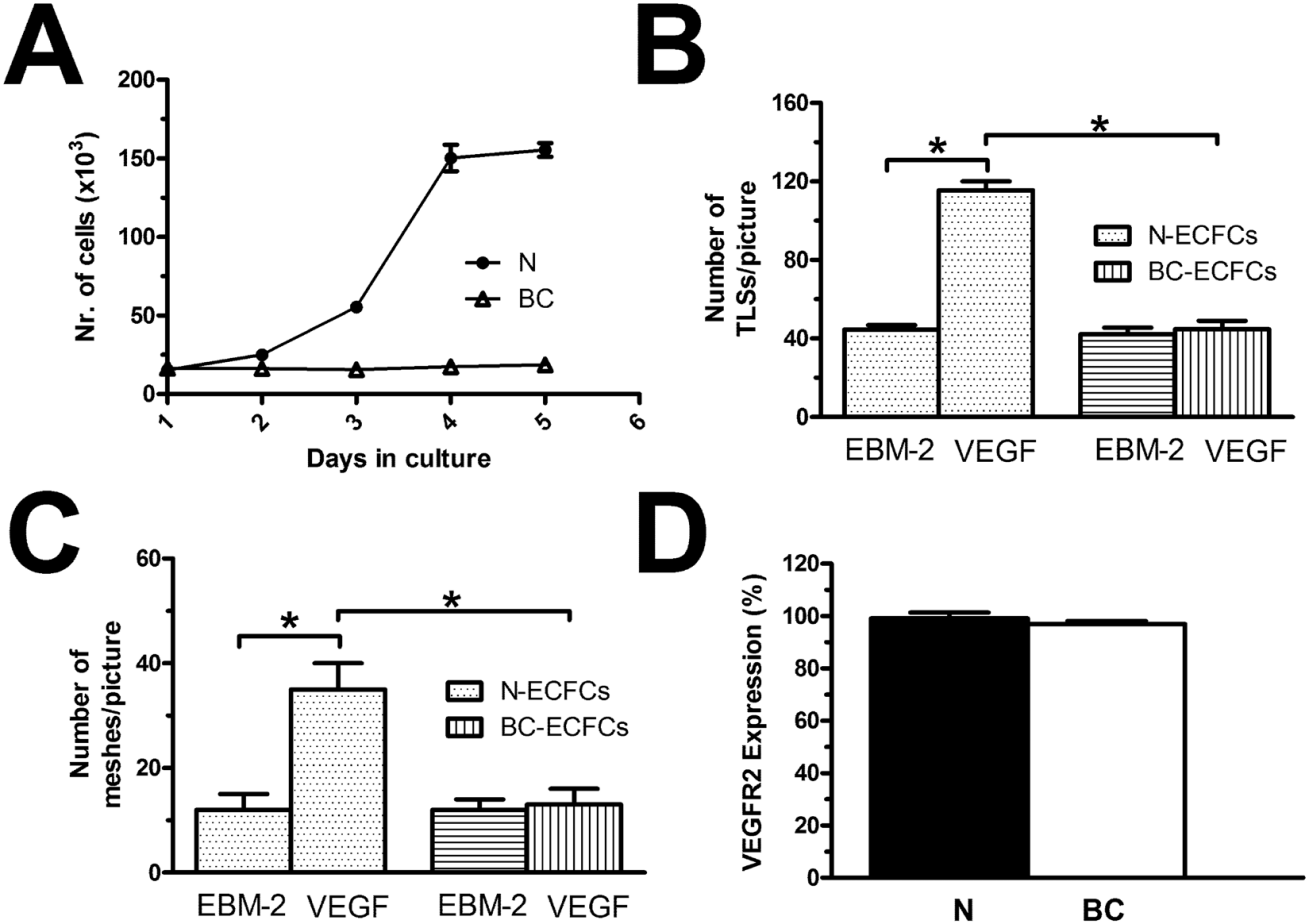
VEGF does not stimulate proliferation in breast cancer-derived endothelial colony forming cells **(A)**, mean±SE of ECFCs counted after five days in culture in the presence of EBM-2 + VEGF (10 ng/mL). The results are representative of three different experiments conducted on cells harvested from three different donors. VEGF stimulated proliferation in N-ECFCs, but not BC-ECFCs. EBM-2 alone, which was used as control, did not induce proliferation either in N- or BC-ECFCs. **(B-C)**, statistical analysis of the dimensional (total TLSs/picture) and topologic (total number of meshes/picture) parameters of the capillary-like network established by N- and BC-ECFCs, respectively, plated in Matrigel scaffolds. *In vitro* angiogenesis was stimulated by plating the cells in the presence of the EBM-2 medium (which is devoid of growth factors) supplemented with VEGF (10 ng/mL), while EBM-2 alone was used as a control. The results are representative of three different experiments conducted on ECFCs deriving from three different donors. The asterisk indicates p<0.05. **(D)**, mean±SE of the percentage of ECFCs expressing VEGFR-2, as assessed by flow cytometry. There was no statistically relevant difference between N- and BC-ECFCs.

We have widely shown that 10 ng/mL is the most effective concentration to promote VEGF-dependent proliferation and *in vitro* tubulogenesis in ECFCs [26, 28, 29, 37]]. Both N- and BC-ECFCs were plated in the presence of EBM-2 supplemented with 2% foetal calf serum and 10 ng/mL VEGF, as described elsewhere [26]. Under such conditions, N-ECFCs reached confluence after 5 days in culture, while BC-ECFCs did not undergo any significant (p<0.05) increase in the rate of cell growth (Figure 2A). Neither N-ECFCs nor BC-ECFCs replicated in the absence of VEGF, while serum starvation did not affect the proliferative response to VEGF in N-ECFCs (data not shown). We further investigated the physiological outcome of VEGF by carrying out a tube formation assay in Matrigel, which reconstitutes the basement membrane extracellular matrix. This assay recapitulates many steps of the angiogenic process, including adhesion, migration, protease activity, and tubule formation, and is, therefore, extremely useful to evaluate whether VEGF differently affects N- and BC-ECFCs [22, 24, 26]. Ten ng/mL VEGF stimulated N-ECFCs to assemble into an extended bidimensional capillary-like network, while it was ineffective on BC-ECFCs (Figure 2B and Figure 2C). These data clearly show that VEGF stimulates proliferation and *in vitro* tubulogenesis in N-ECFCs, but not BC-ECFCs. In order to assess whether the distinct effect of VEGF was associated to the down-regulation of VEGFR-2 in tumor-associated cells, we analyzed its expression in both types of cells through flow cytometry, as shown in [24]. However, no significant difference was observed between N- and BC-ECFCs, as depicted in Figure 2D and in Supplementary Figure 1. Therefore, the inability of VEGF to stimulate proliferation in BC-ECFCs is likely to involve the downregulation of VEGFR2 signalling in tumor-associated ECFCs [13, 23].

### VEGF-induced intracellular Ca^2+^ oscillations are down-regulated in BC-ECFCs

**Figure 3:**
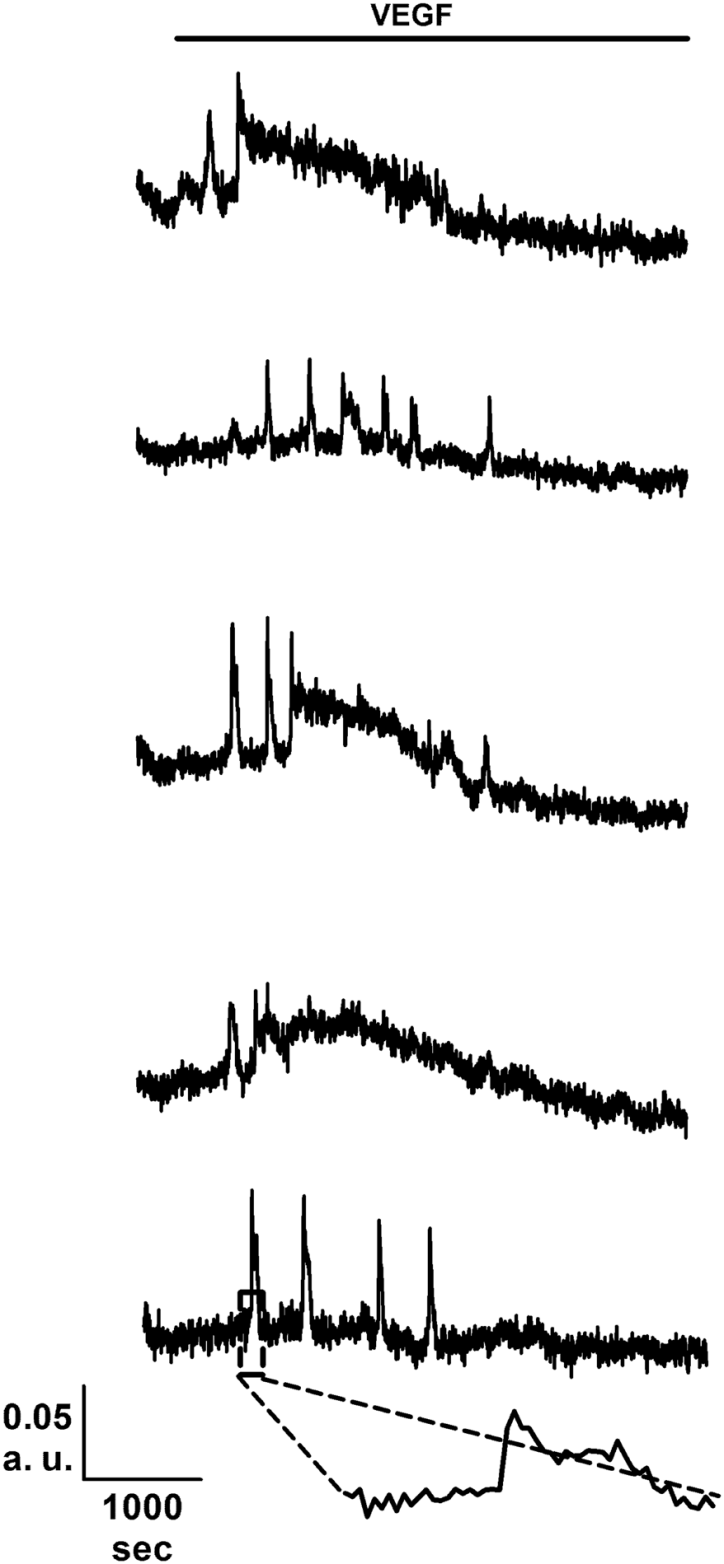
VEGF induces intracellular Ca^2+^ oscillations in breast cancer-derived endothelial colony forming cells VEGF (10 ng/mL) elicits heterogeneous repetitive Ca^2+^ transients in five BC-ECFCs recorded from the same microscopic field. The dashed box in the lowermost trace marks the pacemaker increase in [Ca^2+^]_i_ that features InsP_3_-dependent Ca^2+^ spikes. In these and the other figures, VEGF has been administrated during the time period indicated by the black bars placed above the Ca^2+^ traces.

**Figure 4:**
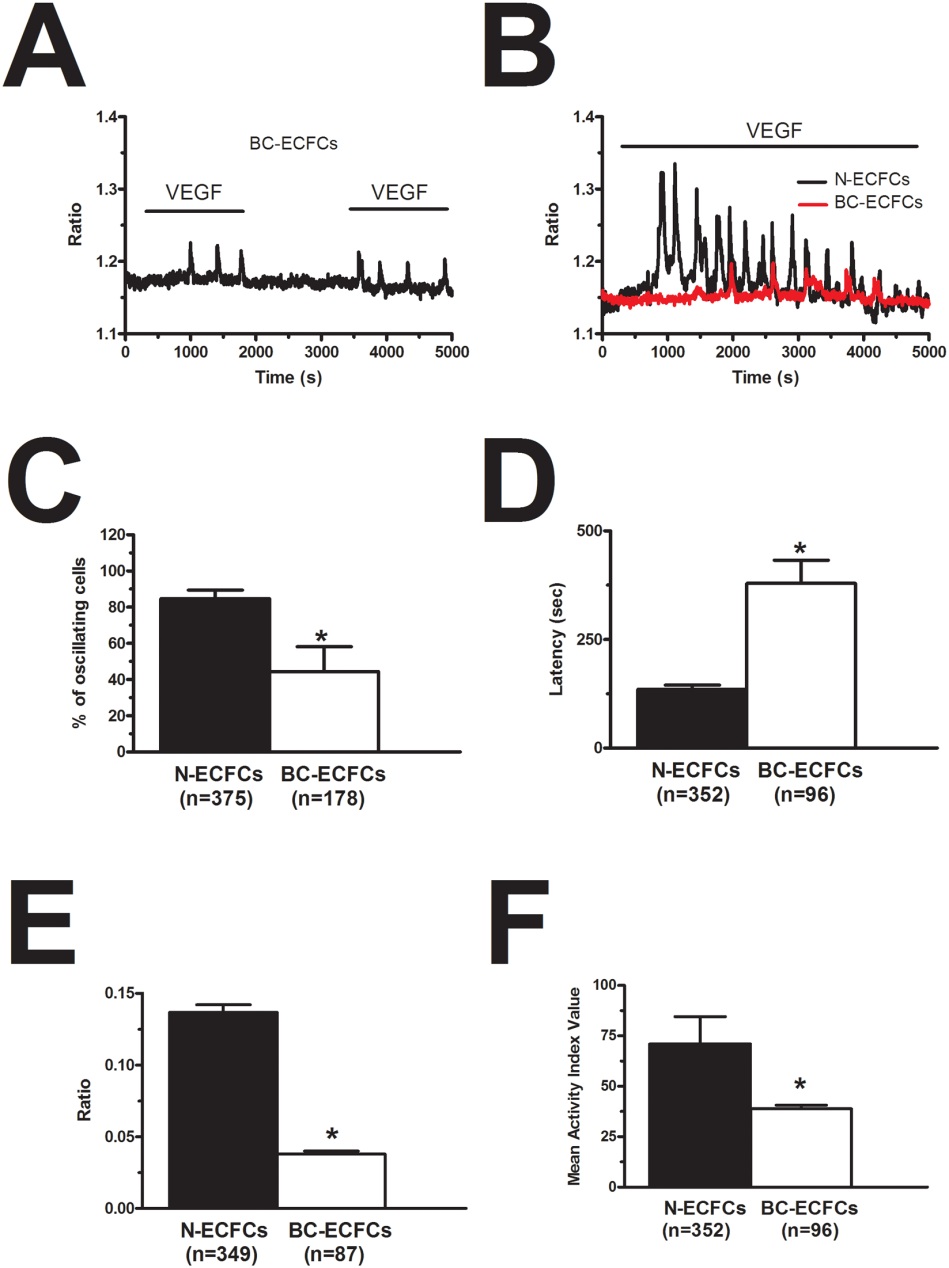
VEGF-induced intracellular Ca^2+^ oscillations are weaker in breast cancer-derived endothelial colony forming cells **(A)**, VEGF (10 ng/mL) removal from the bath reversibly inhibited the Ca^2+^ response to VEGF in BC-ECFCs. **(B)**, VEGF-induced intracellular Ca^2+^ oscillations in N- and BC-ECFC. VEGF was applied at 10 ng/mL to both cell types. In the following panels, bar histograms have been used to compare the fraction of oscillating cells **(C)**, the latency to the first spike **(D)**, the magnitude of the initial Ca^2+^ transient **(E)** and the oscillatory index **(F)** between N- and BC-ECFCs. The asterisk indicates p<0.05.

As mentioned earlier, the mitogenic effect of VEGF impinges on the onset of intracellular Ca^2+^ oscillations, which develop with some delay after addition of the growth factor [26, 28, 29]. Consistent with our preliminary results [22], 10 ng/mL VEGF triggered a detectable increase in [Ca^2+^]_i_ also in BC-ECFCs (Figure 3), while there was no spontaneous Ca^2+^ activity in absence of the agonist (not shown). The percentage of responding cells was 36.9±10.2% (n=477), which was significantly lower (p=0.00339) as compared to that measured in N-ECFCs (99.3±0.7%, n=380). The Ca^2+^ response recorded in BC-ECFCs consisted in either transient Ca^2+^ peaks or short, asynchronous Ca^2+^ oscillations, which did not arise in phase among the neighbouring cells from a given microscopic field (Figure 3). This feature represents the hallmark of VEGF-induced Ca^2+^ oscillations in both ECFCs [27] and mature endothelial cells [38]. Each baseline Ca^2+^ transient was preceded by a slow increase in [Ca^2+^]_i_ (see inset in Figure 3), which is known as pacemaker Ca^2+^ rise and is indicative of InsP_3_-dependent Ca^2+^ release during the spiking response to VEGF [28]. The oscillating response to VEGF was reversibly abolished by removing the agonist from the perfusate (Figure 4A). When compared to N-ECFCs (Figure 4B), a careful statistical analysis revealed that the percentage of oscillating cells (Figure 4C) and the amplitude (Figure 4E) of the first Ca^2+^ transient were significantly (p<0.05) smaller in BC-ECFCs as compared to healthy cells. Conversely, the latency of the Ca^2+^ response was significantly (p<0.05) longer in BC-ECFCs (Figure 4D). We then exploited a recently described home-made software based on wavelet analysis to extract information encoded within the complex spatio-temporal pattern of Ca^2+^ spikes and obtain a straightforward quantitative evaluation of the differences between VEGF-induced Ca^2+^ oscillations in N- and BC-ECFCs [26, 39, 40]. This analysis confirmed that the spiking response to VEGF was significantly (p>0.05) reduced in BC-ECFCs (Figure 4F). Taken together, these data suggest that the down-regulation of intracellular Ca^2+^ oscillations underpins the little, if any, pro-angiogenic effect of VEGF in BC-ECFCs.

### VEGF-induced intracellular Ca^2+^ oscillations require InsP_3_-dependent Ca^2+^ release and SOCE in BC-ECFCs

**Figure 5:**
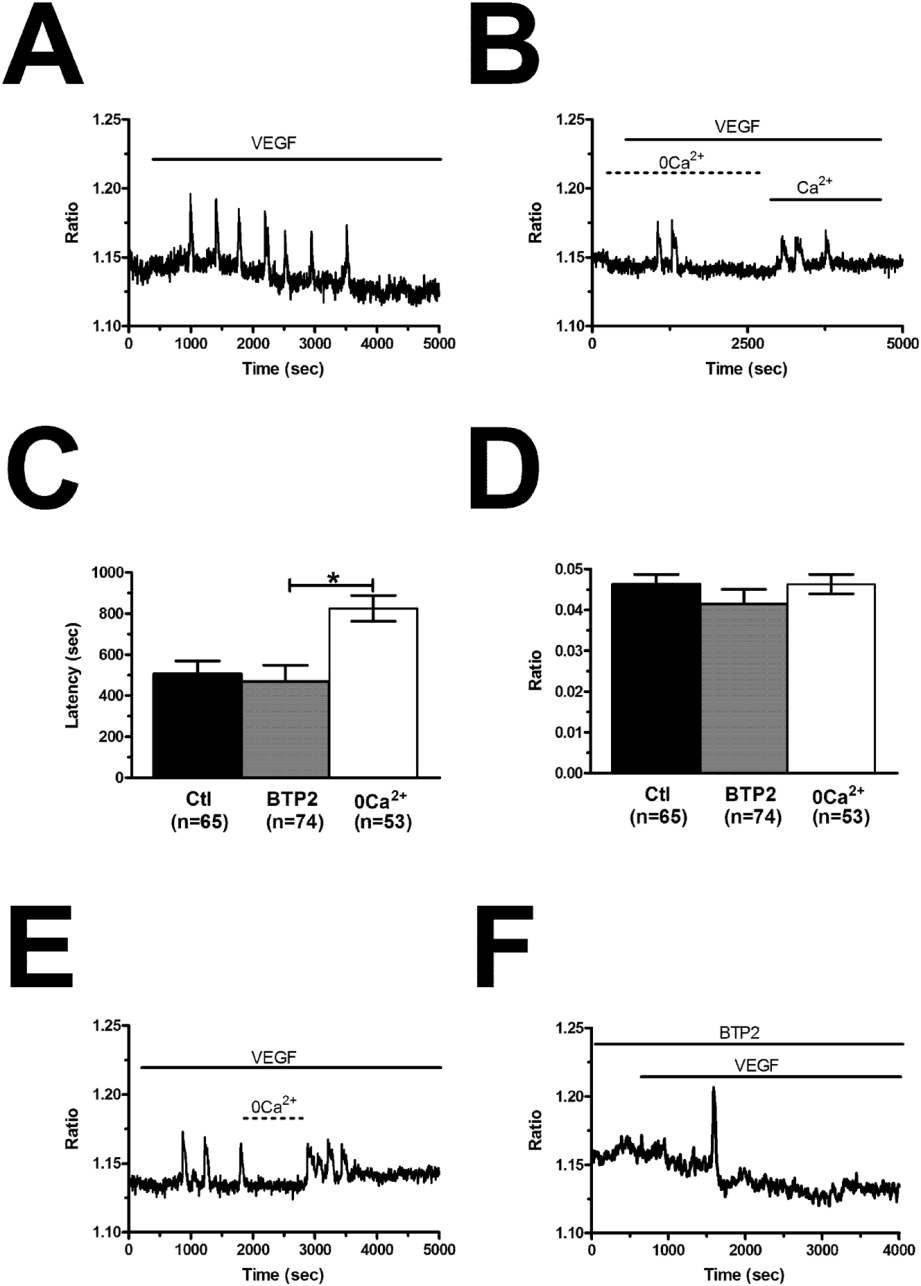
VEGF-induced intracellular Ca^2+^ oscillations are triggered by endogenous Ca^2+^ release and maintained by store-operated Ca^2+^ entry **(A)**, intracellular Ca^2+^ oscillations evoked by VEGF (10 ng/mL) in the presence of extracellular Ca^2+^. **(B)**, VEGF induced only 2 Ca^2+^ spikes in the absence of extracellular Ca^2+^ (0Ca^2+^), whereas Ca^2+^ oscillations resumed upon Ca^2+^ re-addition to the extracellular solution. In the following panels, bar histograms have been used to compare the latency to the first spike **(C)** and the magnitude of the initial Ca^2+^ transient **(D)** under 0Ca^2+^ conditions or preincubation with BTP2 (20 μM, 30 min), which selectively inhibits SOCE in ECFCs. The asterisk indicates p<0.05. **(E)**, removal of extracellular Ca^2+^ (0Ca^2+^) caused a reversible inhibition of ongoing VEGF-induced Ca^2+^ oscillations. VEGF was administered at 10 ng/mL. **(F)**, BTP2 (20 μM, 30 min), a selective SOCE inhibitor, did not prevent the onset of VEGF-induced intracellular Ca^2+^ oscillations, but curtailed their duration to 1-2 transients.

**Figure 6:**
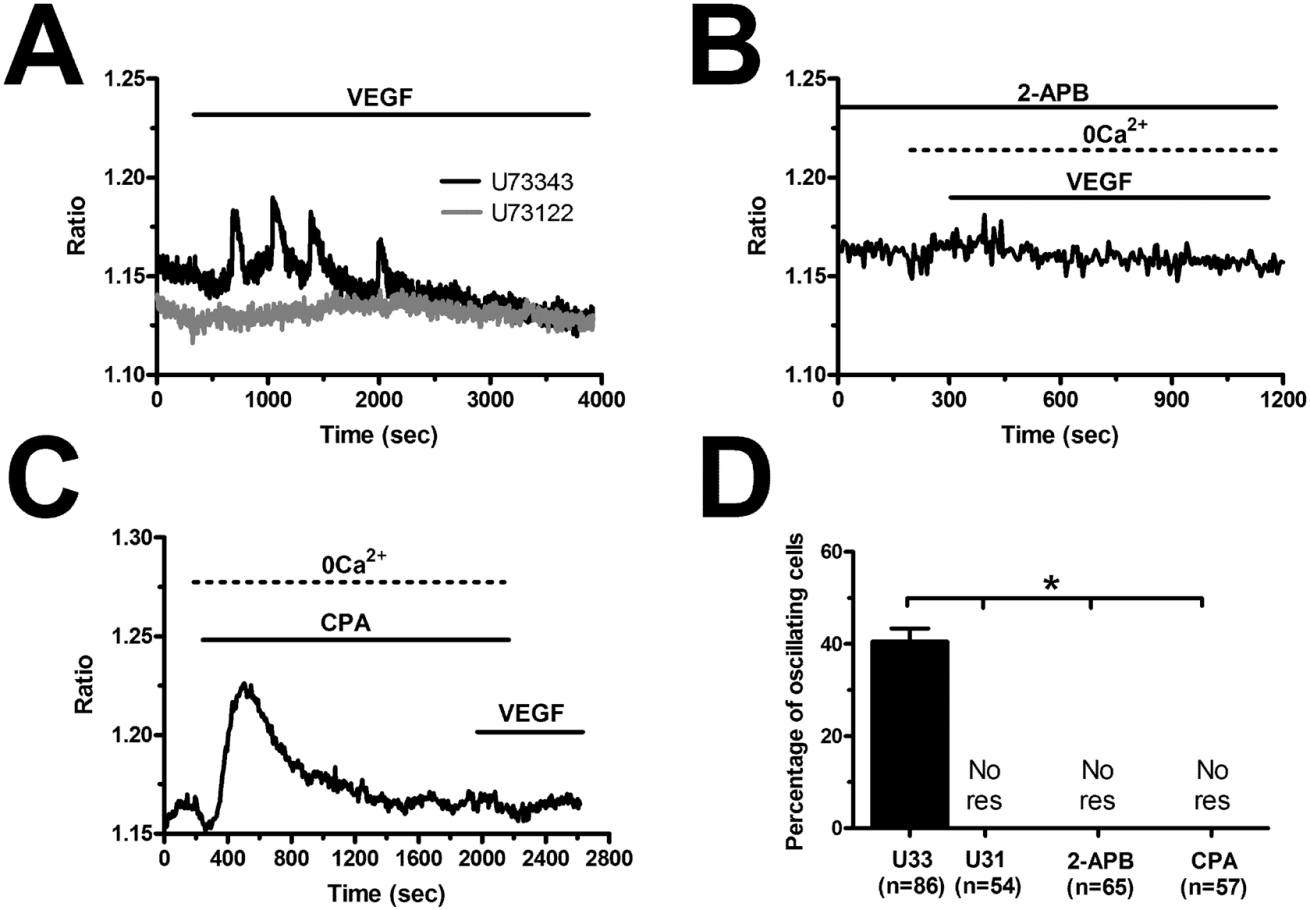
VEGF-induced intracellular Ca^2+^ oscillations are initiated by the PLCγ/InsP_3_ pathway in breast cancer-derived endothelial colony forming cells **(A)**, U73122 (10 μM, 10 min), a selective PLC blocker, prevented the Ca^2+^ response to VEGF, while U73343 (10 μM, 10 min), an inactive analogue of U7322, was without effect. **(B)**, 2-APB (50 μM, 20 min), an InsP_3_R blocker, suppressed VEGF-induced Ca^2+^ oscillations under 0Ca^2+^ conditions to prevent any contaminating effect on plasmalemmal channels. **(C)**, the depletion of the InsP_3_-sensitive ER Ca^2+^ store with CPA (10 μM) in the absence of external Ca^2+^ (0Ca^2+^) prevented the following Ca^2+^ response to VEGF (10 ng/mL). Note the transient increase in [Ca^2+^]_i_ caused by CPA due to the depletion of the ER Ca^2+^ pool. **(D)**, mean±SE of the percentage of BC-ECFCs responding to VEGF (10 ng/mL) under the designated treatments. The asterisk indicates p<0.05. NoR: No response.

The spiking response to VEGF is shaped by the concerted interplay between InsP_3_-dependent Ca^2+^ release and SOCE in N-ECFCs [28], however, this mechanism is subtly remodelled in PMF-derived cells [26]. In order to decipher the molecular underpinnings of VEGF-induced Ca^2+^ oscillations in BC-ECFCs, we first challenged the cells with the growth factor upon removal of Ca^2+^ from the perfusate (0Ca^2+^). Unlike cells bathed in the presence of extracellular Ca^2+^ (Figure 5A), VEGF still induced 1-2 Ca^2+^ transients in the absence of extracellular Ca^2+^, but the Ca^2+^ activity rapidly subsided despite for the prolonged exposure to the agonist (Figure 5B). The latency of the first Ca^2+^ transient was significantly (p<0.05) longer under 0Ca^2+^ conditions (Figure 5C), while there was no statistically relevant difference in its amplitude (Figure 5D). Ca^2+^ oscillations readily resumed upon Ca^2+^ restitution to the bath, thereby showing that the Ca^2+^ signal was initiated by Ca^2+^ mobilization from the intracellular stores and sustained by Ca^2+^ entry across the plasma membrane (Figure 5B). In agreement with this notion, removal of external Ca^2+^ reversibly suppressed ongoing VEGF-induced Ca^2+^ oscillations (Figure 5E). Therefore, we then focussed on the PLCγ/InsP_3_ signalling pathway, which underlies periodic ER Ca^2+^ release in N-ECFCs [28]. VEGF failed to ignite the Ca^2+^ train in the presence of U73122 (10 μM, 10 min) (1-[6-[[(17b)-3-methoxyestra-1,3,5(10)-trien-17-yl]amino]hexyl]-1H-pyrrole-2,5-dione) (Figure 6A), an aminosteroid which selectivity inhibits PLC activity in ECFCs [26, 28, 29], while its inactive structural analogue U73343 (10 μM, 10 min) failed to block the Ca^2+^ response (Figure 6A). Likewise, 2-aminoethoxydiphenyl borate (2-APB; 50 μM, 20 min), a widely employed blocker of InsP_3_ receptors (InsP_3_Rs), suppressed VEGF-induced Ca^2+^ oscillations (Figure 6B). As 2-APB may also interfere with Stim1, Orai and TRP Vanilloid (TRPV) channels [36], these experiments were performed under 0Ca^2+^ conditions to prevent any contaminating effect from Ca^2+^ influx [28]. Finally, VEGF was applied after depletion of the ER Ca^2+^ pool with cyclopiazonic acid (CPA), which selectively inhibits the activity of Sarco-Endoplasmic Reticulum Ca^2+^-ATPase (SERCA), thereby preventing Ca^2+^ sequestration and emptying the endogenous Ca^2+^ stores [28]. As shown in Figure 6C, VEGF failed to increase intracellular Ca^2+^ levels following 30 min preincubation with CPA in the absence of extracellular Ca^2+^ (0Ca^2+^). The statistical analysis of these data has been synthesized in Figure 6D. SOCE represents the most important pathway for Ca^2+^ entry in N-ECFCs [27, 37]. Therefore, we assessed its contribution to the maintenance of VEGF-induced Ca^2+^ oscillations by pre-incubating the cells with BTP2 (N-(4-[3,5-bis(trifluoromethyl)-1H-pyrazo l-1-yl]phenyl)-4-methyl-1,2,3-thiadiazol e-5-carboxamide) (20 μM, 30 min), a pyrazole derivative which selectively inhibits SOCE in both ECFCs [31, 37] and a growing number of cell types [41, 42]. This treatment did not prevent the onset of the Ca^2+^ response to VEGF, but curtailed its duration to 1-2 Ca^2+^ spikes (Figure 5F), thereby mimicking the effect of 0Ca^2+^. Unlike 0Ca^2+^ conditions, however, BTP2 did not affect the latency of the first Ca^2+^ transient (Figure 5C), as well as it did not reduce its amplitude (Figure 5D). Overall, these observations clearly show that VEGF-induced Ca^2+^ oscillations required the InsP_3_-dependent rhythmical ER Ca^2+^ discharge and were sustained by SOCE also in BC-ECFCs. Therefore, the downregulation of the Ca^2+^-dependent pro-angiogenic response to VEGF in these cells must involve the remodeling of one or more components of their Ca^2+^ toolkit.

### The ER Ca^2+^ content is decreased, while SOCE is unaffected, in BC-ECFCs

**Figure 7:**
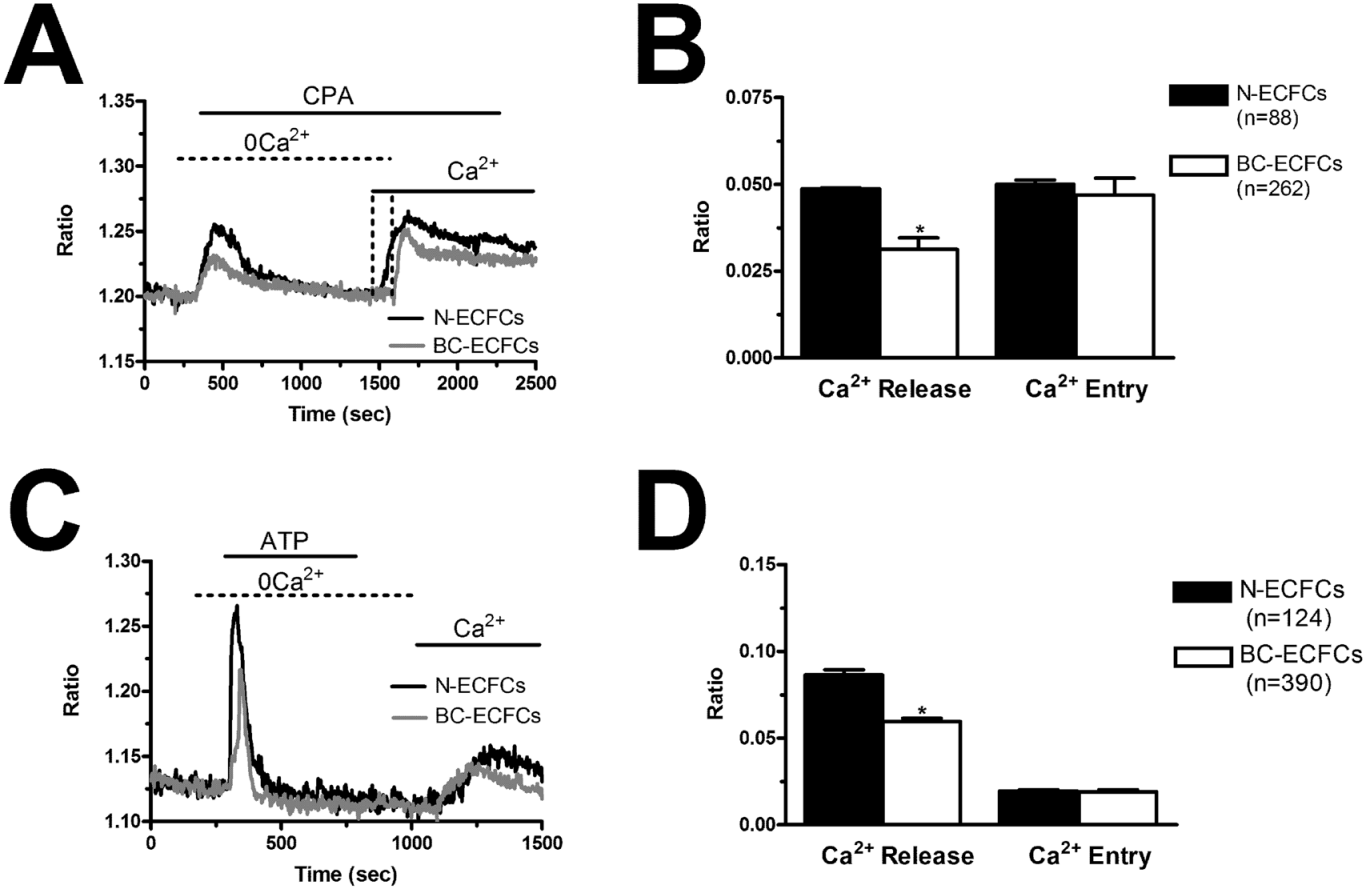
Remodelling of the Ca^2+^ toolkit in breast cancer-derived endothelial colony forming cells **(A)**, the intracellular Ca^2+^ pool was depleted by stimulating the cells with CPA (10 μM) in the absence of external Ca^2+^ (0Ca^2+^), and Ca^2+^ influx through store-operated channels was then assessed on Ca^2+^ replenishment to the bathing solution. **(B)**, mean±SE of the amplitude of CPA-induced Ca^2+^ release and CPA-induced SOCE in N- and BC-ECFCs. The asterisk indicates p<0.05. **(C)**, ATP (100 μM) evoked a transient increase in [Ca^2+^]_i_ in N- and BC-ECFCs bathed in the absence of external Ca^2+^ (0Ca^2+^). **(D)** ATP was then removed from the bath, while Ca^2+^ was readded to the perfusate in order to measure SOCE. F, mean±SE of the amplitude of ATP-induced Ca^2+^ release and ATP-induced SOCE recorded from both N- and BC-ECFCs. The asterisk indicates p<0.05.

In order to assess whether and how the intracellular Ca^2+^ handling is altered in BC-ECFCs, we exploited the “Ca^2+^ add-back” protocol, which consists in first depleting the ER Ca^2+^ pool with CPA (10 μM) in the absence of extracellular Ca^2+^ (0Ca^2+^) and then restoring extracellular Ca^2+^ to monitor the following SOCE [24, 25]. This protocol has been largely used to assess both the ER Ca^2+^ content and the extent of SOCE activation in a myriad of cancer cells [43, 44], including tumor-associated ECFCs [26, 39, 40]. We found that CPA-induced ER Ca^2+^ release was significantly (p<0.05) reduced as compared to N-ECFCs, while SOCE amplitude was unaffected (Figure 7A and Figure 7B). To further corroborate these data, we repeated the “Ca^2+^ add-back” protocol in the presence of the physiological autacoid, ATP (100 μM), which binds to metabotropic P2Y receptors to stimulate InsP_3_ synthesis and promote InsP_3_-dependent ER Ca^2+^ release [26, 39, 40]. Again, ATP-induced InsP_3_-dependent ER Ca^2+^ release was significantly (p<0.05) lower in BC-ECFCs, while ATP-induced SOCE was unaltered as respect to N-ECFCs (Figure 7C and Figure 7D). As previously described [24], ATP was removed from the extracellular solution 100 sec before Ca^2+^ re-addition to prevent Ca^2+^ entry across store-independent pathways (Figure 7C). The reduction in ATP-induced intracellular Ca^2+^ release was not due to the down-regulation of InsP_3_Rs, as qRT-PCR analysis carried out by using the specific primers described in Supplementary Table 1 showed that there was no statistically relevant difference in the expression of all InsP_3_R isoforms between N- and BC-ECFCs (Supplementary Figure 2). Collectively, these data concur with the preliminary data we recently reported [22] and strongly suggest that the Ca^2+^ signalling toolkit is partially remodelled in BC-ECFCs. This dysregulation consists in a dramatic drop in ER Ca^2+^ levels, which might prevent the InsP_3_-dependent ER Ca^2+^ cycling that underlies the pro-angiogenic response to VEGF.

### The pharmacological profile and molecular composition of SOCE is similar to that described in N-ECFCs

**Figure 8:**
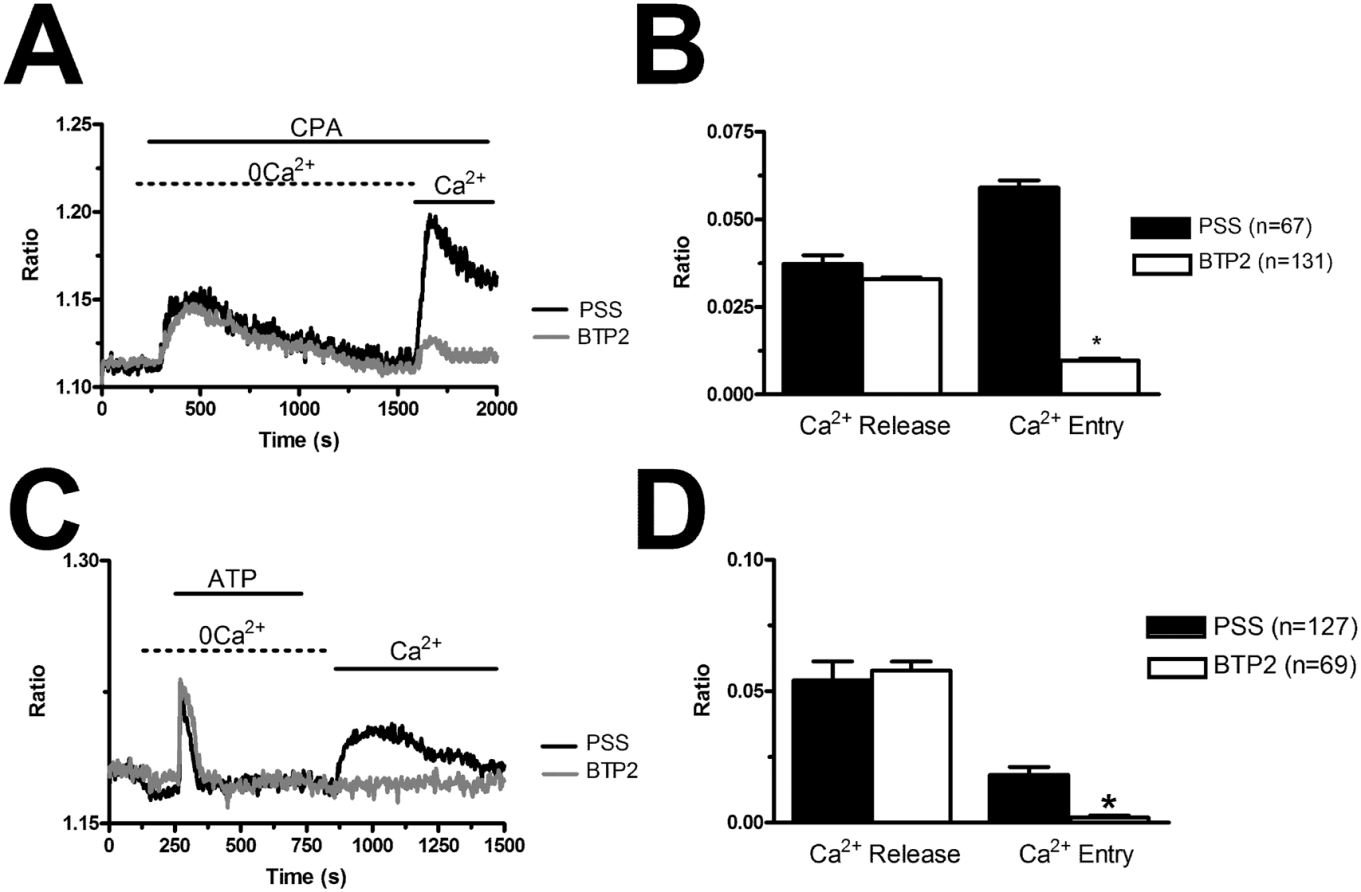
BTP2 inhibits store-dependent Ca^2+^ entry in breast cancer-derived endothelial colony forming cells **(A)**, CPA-elicited SOCE in the absence and presence of BTP2 (20 μM). The cells were pre-incubated with the drug for 30 min before the beginning of the experimental protocol. CPA was administered at 10 μM. **(B)**, mean±SE of the amplitude of CPA-induced intracellular Ca^2+^ release and CPA-induced SOCE in the absence and presence of BTP2. The asterisk indicates p<0.05. **(C)**, ATP-evoked intracellular Ca^2+^ release and SOCE in the presence and absence of BTP2 (20 μM). The cells were pre-incubated with the drug for 30 min before the beginning of the experimental protocol. ATP was applied at 100 μM. **(D)**, mean±SE of the amplitude of CPA-induced Ca^2+^ release and CPA-induced SOCE in the absence and presence of BTP2. The asterisk indicates p<0.05.

**Figure 9:**
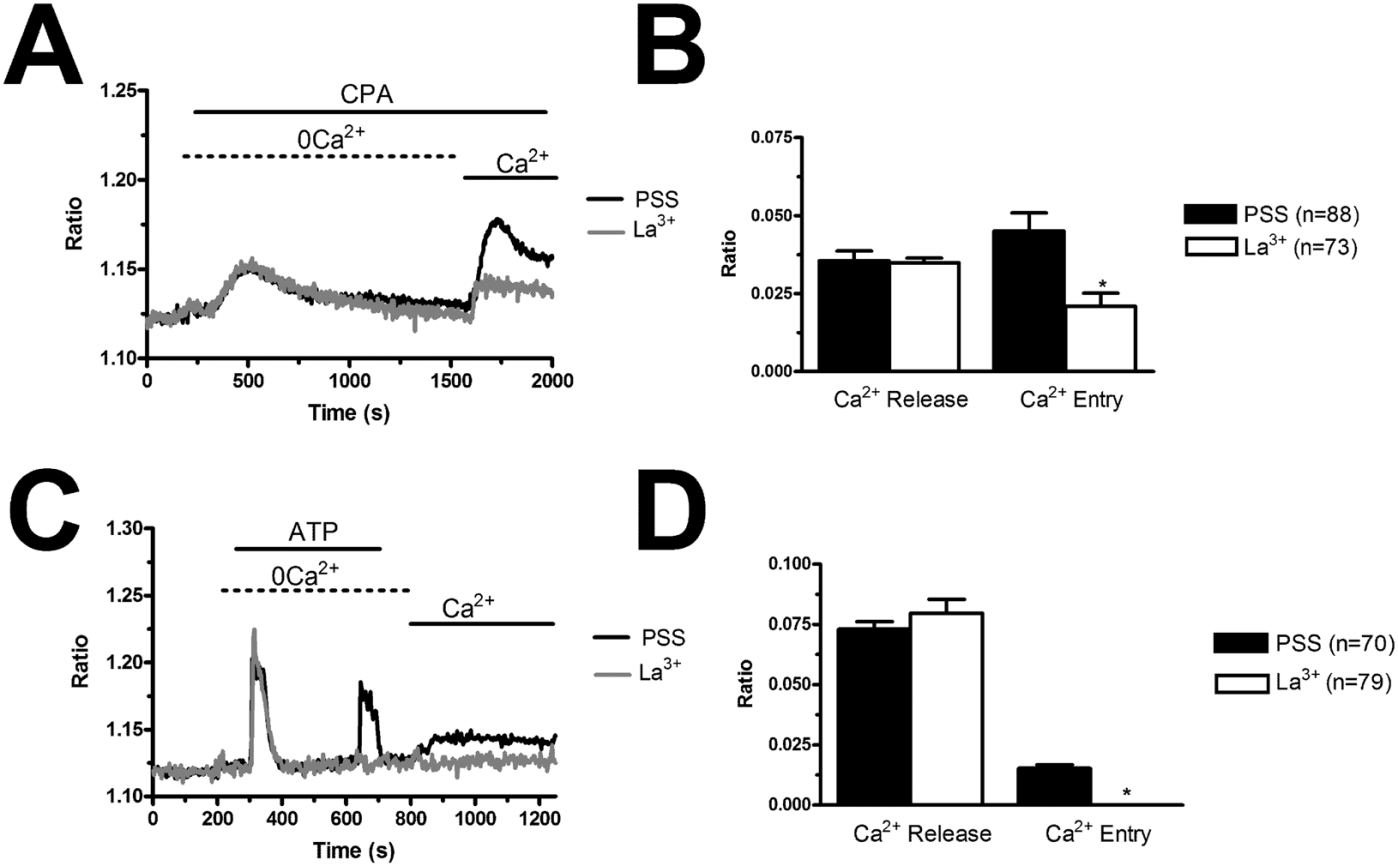
La^3+^ inhibits store-dependent Ca^2+^ entry in breast cancer-derived endothelial colony forming cells **(A)**, CPA-elicited SOCE in the absence and presence of La^3+^ (10 μM). The cells were pre-incubated with the drug for 20 min before the beginning of the experimental protocol. CPA was administered at 10 μM. **(B)**, mean±SE of the amplitude of CPA-induced intracellular Ca^2+^ release and CPA-induced SOCE in the absence and presence of La^3+^. The asterisk indicates p<0.05. **(C)**, ATP-evoked intracellular Ca^2+^ release and SOCE in the presence and absence of La^3+^ (10 μM). The cells were pre-incubated with the drug for 30 min before the beginning of the experimental protocol. ATP was applied at 100 μM. **(D)**, mean±SE of the amplitude of CPA-induced Ca^2+^ release and CPA-induced SOCE in the absence and presence of BTP-2. The asterisk indicates p<0.05.

**Figure 10:**
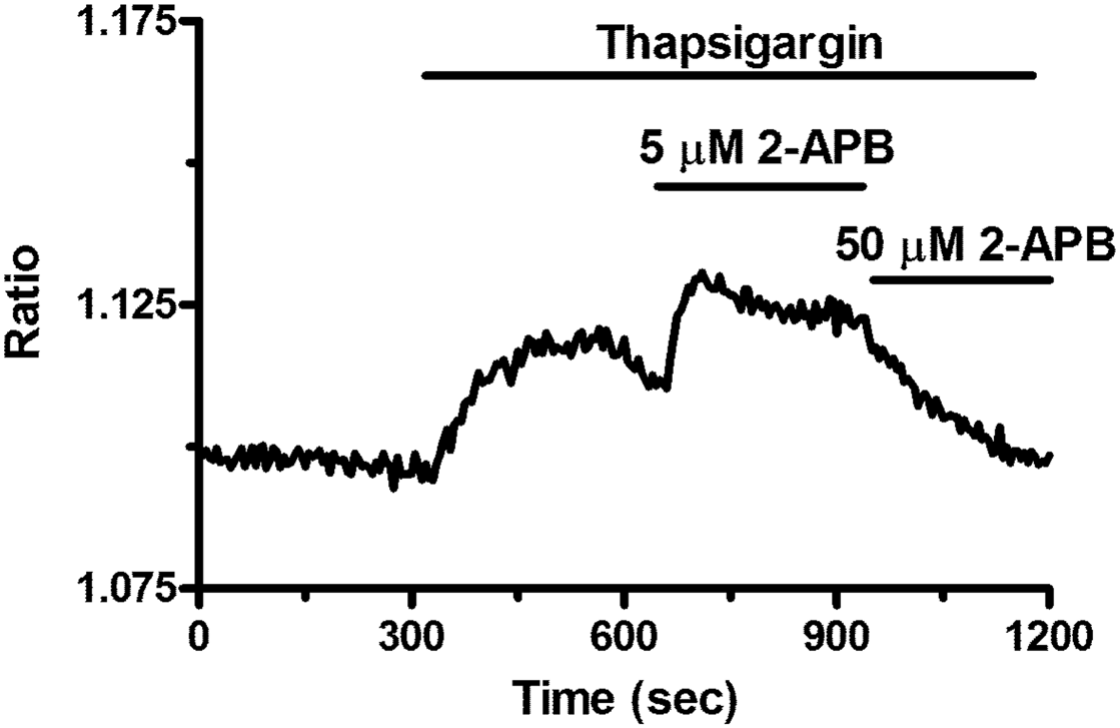
2-APB exerts a dose-dependent effect on store-operated Ca^2+^ entry in breast cancer-derived endothelial colony forming cells 5 μM 2-APB enhanced SOCE induced by thapsigargin (2 μM), whereas 50 μM inhibited it. This is a pharmacological property of Orai1 containing store-operated channels.

In order to confirm that BTP2 selectively inhibits agonist-induced SOCE in BC-ECFCs, we carried out the “Ca^2+^ add-back” protocol in the presence of this drug. BTP2 (20 μM, 30 min) selectively blocked both CPA- and ATP-induced SOCE, while it did not affect the initial phase of intracellular Ca^2+^ mobilization (Figure 8A–8D). The same effect was achieved by the trivalent cation, La^3+^ (10 μM, 30 min) (Figure 9A–9D). As discussed elsewhere [36, 37, 45], BTP2 and low micromolar doses of lanthanides specifically target store-operated channels (SOCs) whose pore-forming subunits are provided by Orai1 and/or TRPC1. These data further corroborate the notion that both passive CPA-facilitated and active InsP_3_-mediated ER Ca^2+^ store depletion led to the activation of the same plasmalemmal Ca^2+^-permeable pathway in ECFCs [23, 27]. Recent studies showed that Orai3 may replace Orai1 as pore-forming of store-operated channels [36, 43, 46]. To assess this issue in BC-ECFCs, we took advantage from the biphasic dependence of Orai1 on 2-APB. 2-APB activates Orai1 at 5 μM, while inhibits it at concentrations higher than 30 μM [24, 36]. Therefore, we fully activated SOCE by challenging BC-ECFCs with thapsigargin (2 μM), another SERCA inhibitor structurally unrelated to CPA, in the presence of extracellular Ca^2+^. As shown in RCC-ECFCs [24], this treatment caused a sustained increase in [Ca^2+^]_i_ which was due to both passive ER Ca^2+^ release and SOCE. The following addition of 5 μM 2-APB caused a further increase in [Ca^2+^]_i_, which was in turn suppressed by the subsequent application of 50 μM 2-APB in 61 cells (Figure 10). This data further corroborates the role of Orai1 in SOCE activation in BC-ECFCs.

**Figure 11:**
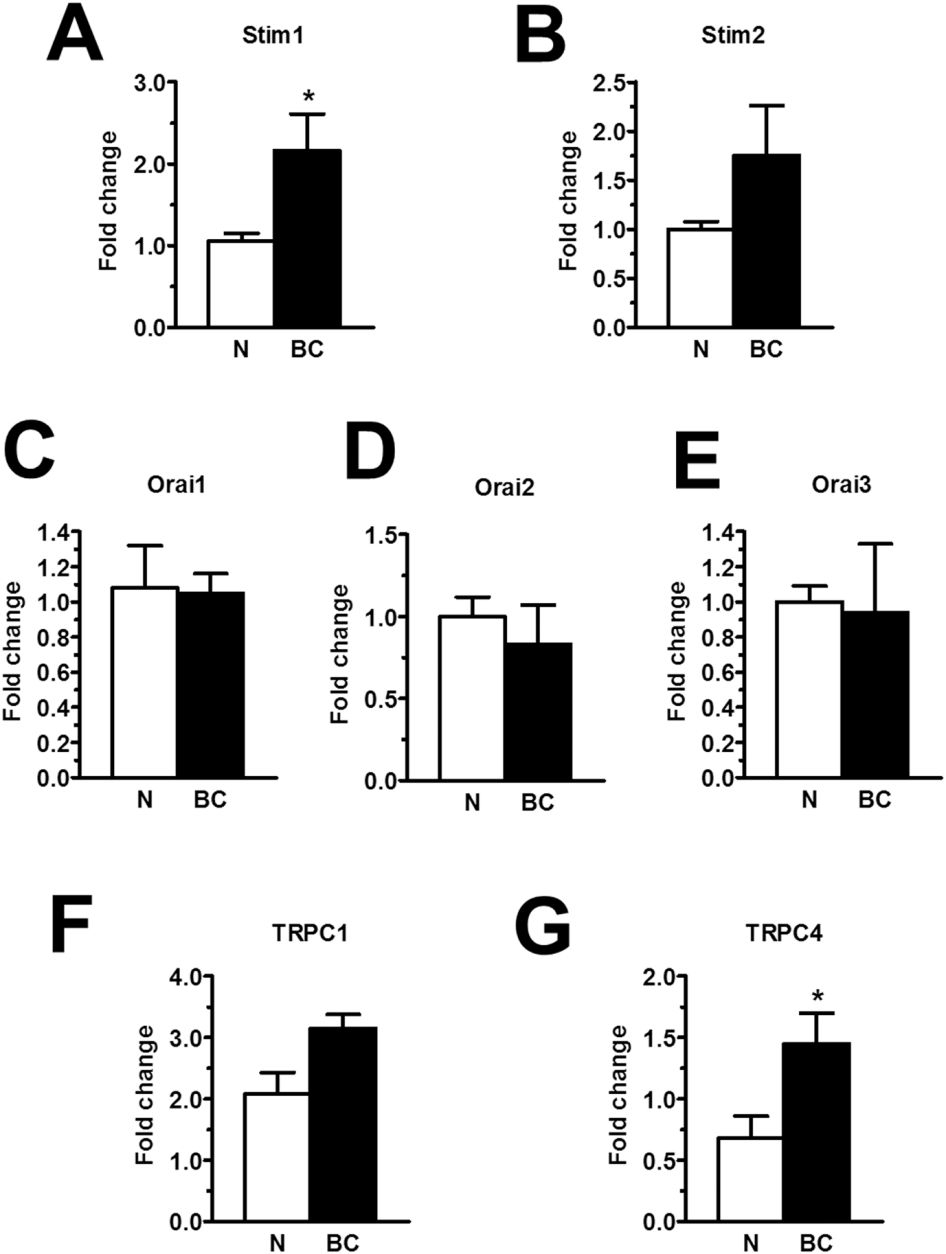
The expression of Stim1-2, Orai1-3, TRPC1 and TRPC4 transcripts in breast cancer-derived endothelial colony forming cells qRT-PCR showing increased expression of Stim1 **(A)** mRNA in BC-ECFCs compared to N-ECFCs. Conversely, Stim2 **(B)**, Orai1 **(C)**, Orai2 **(D)**, Orai3 **(E)**, TRPC1 **(F)**, were not differently expressed in BC-ECFCs. Also, the expression of TRPC4 mRNA was enhanced in BC-ECFCs **(G)**. Bars represent mean±SE of at least 4 different experiments each from different RNA extracts. The asterisk indicates p<0.05 *vs.* N-ECFCs (ANOVA followed by Newman-Keuls’ *Q* test). The PCR products were of the expected size (not shown): Orai1, 257 bp; Orai2, 334 bp; Orai3, 159 bp; Stim1, 347 bp; Stim2, 186 bp; TRPC1, 307 bp and TRPC4, 300 bp.

**Figure 12:**
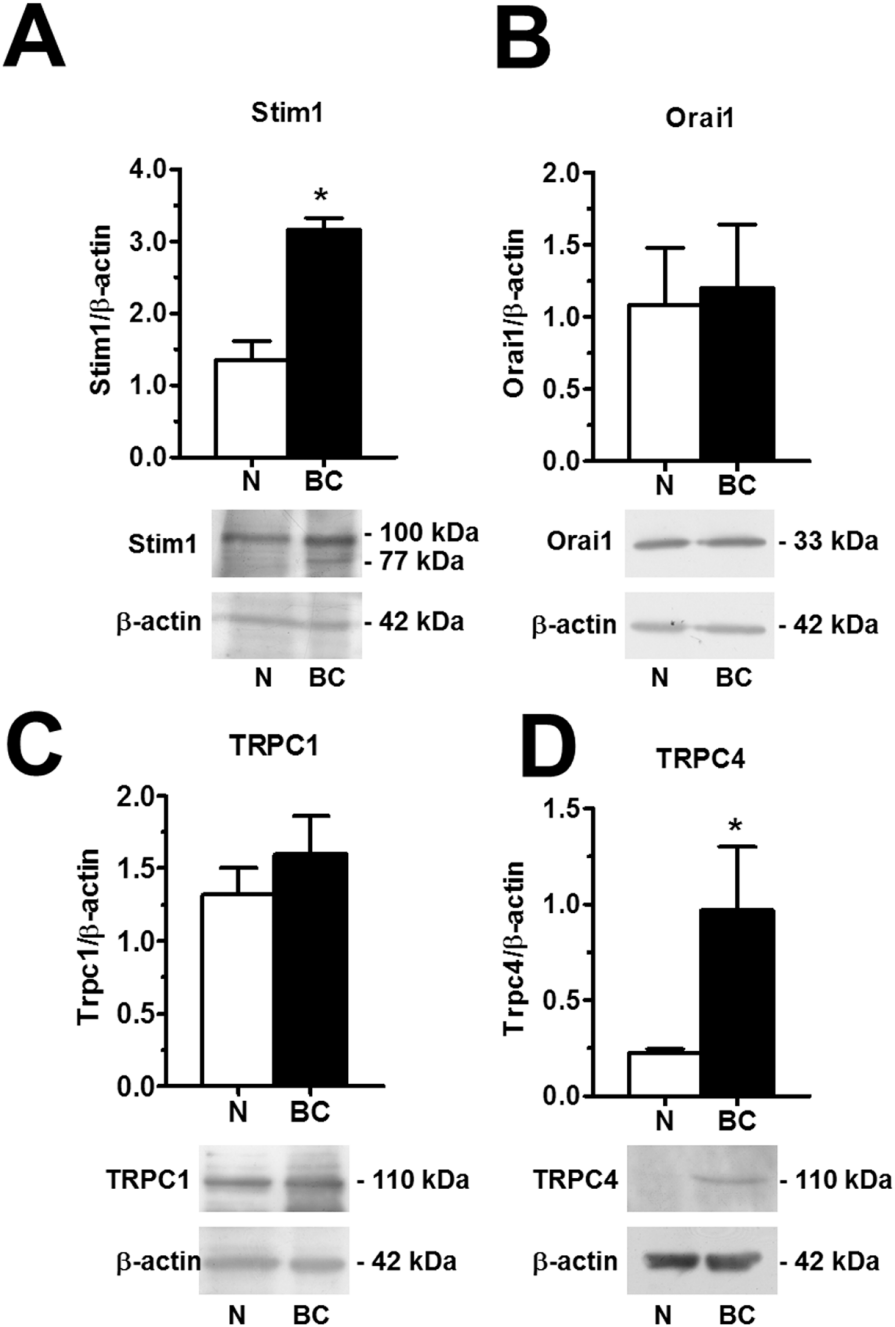
Orai1, Stim1, TRPC1 and TRPC4 proteins are up-regulated in breast cancer-associated endothelial colony forming cells Western blot and densitometry depicting the significant elevation in Orai1 **(A)**, Stim1 **(B)**, TRPC1 **(C)**, and TRPC4 **(D)** proteins in BC-ECFCs as compared to N-ECFCs. Blots for Orai1, Stim1, TRPC1 and TRPC4 representative of 4 different experiments are shown in the lower panel. Lanes were loaded with 20 μg of proteins. Major bands of the expected molecular weight were observed in both cell types. One additional band of 77 kDa was detected by anti-Stim1 in RCC-EPCs. When both Stim1 bands (77 and 100 kDa) were compared to the single band detected at 100 kDa in N-ECFCs, the expression of Stim1 protein became significantly higher in BC-ECFCs. Each bar in the upper panel represents the mean±SE of the densitometric analysis of four different experiments. The asterisk indicates p<0.01 (Student's *t*-test).

To extend this information at molecular level, we investigated the expression of the molecular components of SOCE through both qRT-PCR and immunoblotting. The expression of Stim1-2, Orai1-3, TRPC1-7 transcripts was assessed by qRT-PCR analysis of mRNA extracts from N- and BC-ECFCs, as previously shown [24, 25, 47]. We utilized the specific primers described in Supplementary Table 2, whereas negative controls were conducted by omitting the reverse transcriptase (not shown). The housekeeping gene β-actin served as reference gene for data normalization. We found that only Stim1 (Figure 11A) was significantly (p<0.05) up-regulated in BC-ECFCs as compared to N-ECFCs, while there was no remarkable difference in the pattern of expression of Stim2 (Figure 11B), Orai1 (Figure 11C), Orai2 (Figure 11D) and Orai3 (Figure 11E). Also, TRPC1 mRNA levels did not differ between N- and BC-ECFCs, while TRPC4 was significantly (p<0.05) over-expressed in tumor-associated cells (Figure 11F and Figure 11G). Similar to N-ECFCs, BC-ECFCs lacked TRPC2, TRPC3, TRPC5, TRPC6 and TRPC7 (data not shown). These data were confirmed at protein levels by a Western blot analysis performed by using affinity-purified antibodies directed against Stim1, Orai1, TRPC1 and TRPC4 (Figure 12). Immunoblots revealed a major band of 33 kDa for Orai1 and of 110 kDa for TRPC1 in both cell types, whereas the anti-Stim1 antibody detected two bands of 100 kDa and 77 kDa only in BC-ECFCs. Stim1 was detected as a double also in RCC-ECFCs [24], several human BC and RCC cell lines [43, 48], and primary cultures of human mestatic RCC cell lines [49]. Densitometric analysis of the gels showed that Stim1 protein was up-regulated in BC-ECFCs, while Orai1 and TRPC1 proteins were equally expressed in both cell types. Similar to Stim1, immunoblotting revealed a single band of 110 kDa for TRPC4 (Figure 12D), which was significantly (p<0.05) up-regulated compared to N-ECFCs. Taken together, these findings demonstrate that: 1) the pharmacological profile and molecular composition of SOCE in BC-ECFCs is similar to that described in N-ECFCs; and 2) the attenuation of VEGF-induced Ca^2+^ oscillations is unlikely to involve the recruitment of an alternative SOC pathway in tumor-associated cells. These data also confirm that BTP2 selectively targets SOCE as TRPC3 and TRPC5, that could be inhibited by this drug in heterologous expression systems [36], are absent in BC-ECFCs. Likewise, BC-ECFCs lack diacylglycerol-gated Ca^2+^-permeable channels, such as TRPC3, TRPC6 and TRPC7, which consists with our previous findings in N- and RCC-ECFCs [24].

### The pharmacological blockade of SOCE inhibits BC-ECFC proliferation and *in vitro* tubulogenesis

**Figure 13:**
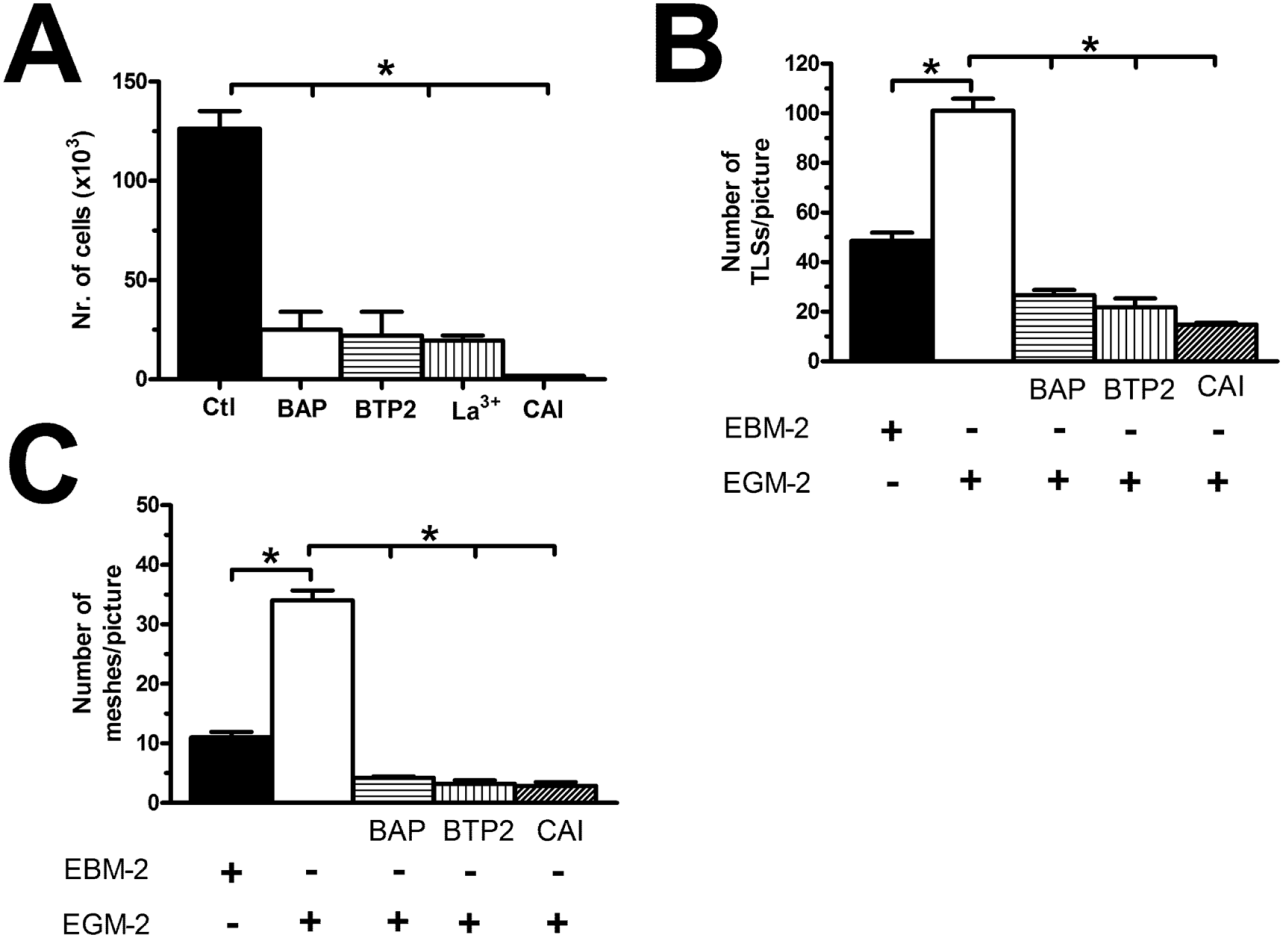
The pharmacological inhibition of store-operated Ca^2+^ entry blocks proliferation in breast cancer-derived endothelial colony forming cells **(A)**, BAPTA (30 μM, 2 hours), a membrane-permeable buffer of intracellular Ca^2+^ levels, BTP2 (20 μM, 30 min), La^3+^ (10 μM, 30 min) and CAI (10 μM, 30 min) blocked proliferation in BC-ECFCs cultured in the presence of EGM-2. The asterisk indicates p<0.05. **(B-C)**, statistical analysis of the dimensional (total TLSs per picture) and topological (total number of junctions between adjacent TLS and total number of meshes per picture) parameters of the capillary-like networks established by BC-ECFCs plated in Matrigel scaffolds in the presence and absence of BAPTA (30 μM, 2 hours), BTP2 (20 μM, 30 min), and CAI (10 μM, 30 min). *In vitro* angiogenesis was stimulated by plating the cells in the presence of EGM-2, while the EBM-2 medium (which is devoid of growth factors) was used as a control. The results are representative of three different experiments conducted on cells derived from three different donors. The asterisk indicates p<0.05.

**Figure 14:**
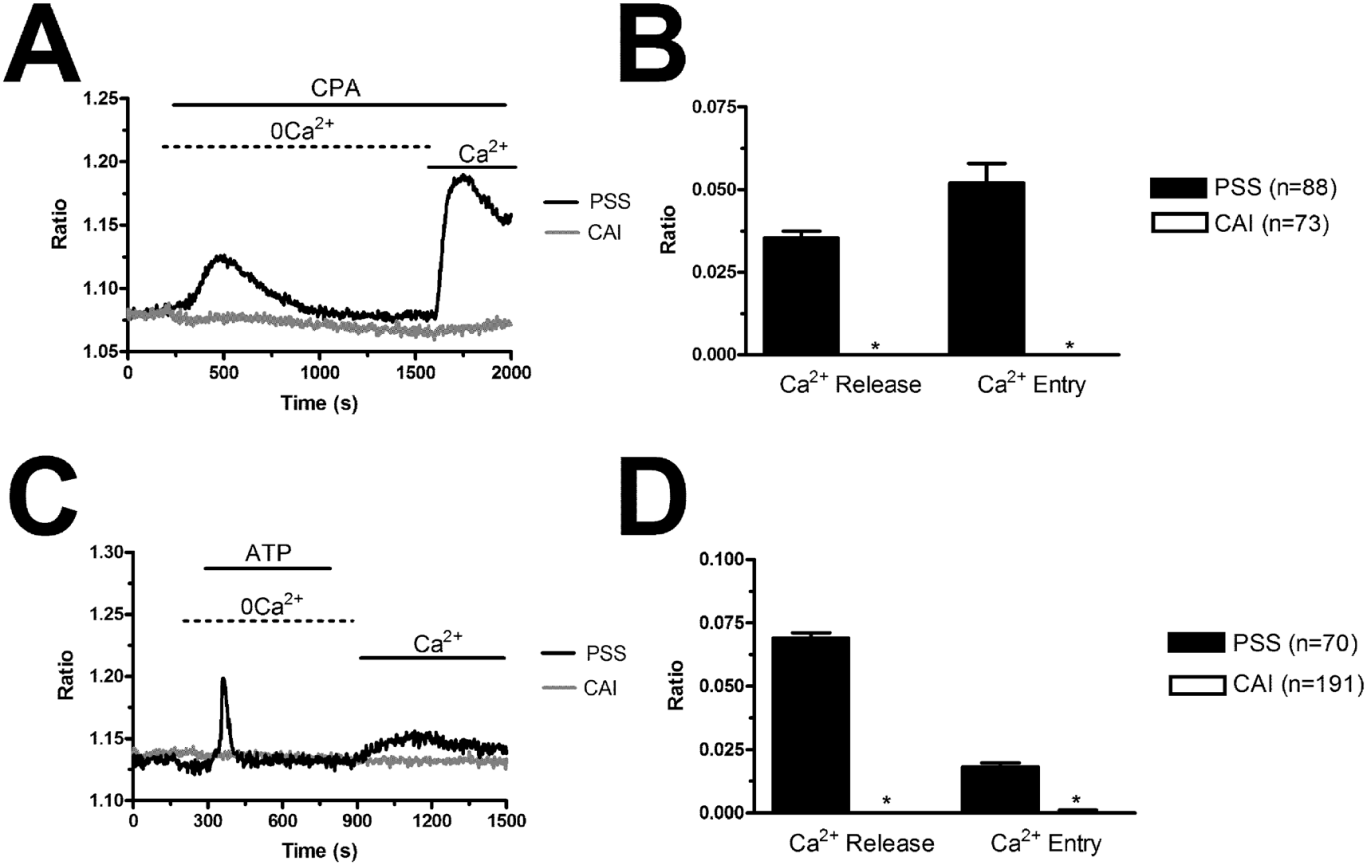
Carboxyamidotriazole suppresses intracellular Ca^2+^ signalling in endothelial progenitor cells **(A)**, CAI (10 μM, 20 min) abolishes the Ca^2+^ response to CPA (10 μM) in BC-ECFCs. **(B)**, mean±SE of the amplitude of CPA-induced intracellular Ca^2+^ release and SOCE in BC-ECFCs. **(C)**, CAI (10 μM, 20 min) abolishes the Ca^2+^ response to ATP (100 μM) in BC-ECFCs. B, mean±SE of the amplitude of ATP-induced intracellular Ca^2+^ release and SOCE in BC-ECFCs. The asterisk indicates p<0.05.

The observation that VEGF does not stimulate proliferation and tube formation in BC-ECFC leads to the quest for alternative targets to halt BC vascularization. Our previous work provided the evidence that SOCE represents a druggable signalling pathway to inhibit the angiogenic activity of tumor-associated ECFCs [24, 25, 47]. Our recent study showed that there was no difference in either growth kinetics or tubulogenic rate between N- and BC-ECFCs cultured in EGM-2 [22]. Therefore, we first ascertained whether BC-ECFC proliferation was inhibited in BC-ECFCs cultured in EGM-2 supplemented with either of the following drugs: BAPTA (30 μM, 1 hour), BTP2 (20 μM, 30 min), and La^3+^ (10 μM, 30 min). As observed earlier for N-, RCC-, and IH-ECFCs, all of those treatments also prevented BC-ECFCs from reaching confluence at 5 days from plating (Figure 13A). Finally, we probed the effect of carboxyamidotriazole (CAI), a synthetic small molecule non-specific inhibitor of various types of Ca^2+^-permeable channels which hastens proliferation in BC endothelial cells [50] and RCC-ECFCs [24]. We first confirmed that CAI (10 μM, 20 min) fully abolished both phases of the Ca^2+^ response to CPA (Figure 14A and Figure 14B) and ATP (Figure 14C and Figure 14D). Then we found that CAI prevented VEGF-induced Ca^2+^ oscillations (Supplementary Figure 3) and blocked proliferation in BC-ECFCs (Figure 13A). Furthermore, we found that BAPTA (30 μM, 1 hour), BTP2 (20 μM, 30 min), and CAI (10 μM, 20 min) also blocked *in vitro* tubulogenesis when BC-ECFCs were plated in Matrigel in the presence of the EGM-2 medium. Again, we evaluated both dimensional (total number of TLSs per picture) (Figure 13B) and topological (number of meshes per picture) (Figure 13C) parameters of the capillary-like network generated by BC-ECFCs plated in a Matrigel scaffold in the presence and the absence of the aforementioned drugs. Therefore, Ca^2+^ signalling in general and, particularly, SOCE could serve as a suitable target to dampen vascularization also in BC.

## DISCUSSION

VEGFR2 has long been regarded as the most suitable target to interfere with tumor vascularization and increase OS in patients affected by highly angiogenic tumors, such as RCC and BC. The majority of *in vitro* studies which led to this assertion were, however, carried out on normal endothelial cell lines, such as human umbilical vein endothelial cells (HUVECs) and human dermal microvascular endothelial cell (HMVECs-D) [24]. Herein, we took advantage from the availability of patients-derived cells to assess whether VEGF stimulates proliferation in ECFCs, which are likely to support the angiogenic switch during BC development [19–21]. Our results provide the first evidence that ECFCs, which contribute to the angiogenic switch in BC [19, 21], are insensitive to VEGF due to the attenuation of pro-angiogenic intracellular Ca^2+^ oscillations that arise upon VEGFR-2 activation in normal cells. These results may help to understand the cellular and molecular underpinnings of primary and secondary refractoriness of BC patients to anti-VEGF therapies.

In stringent disagreement with the traditional assumption [6, 51], VEGF was unable to stimulate proliferation and tube formation in BC-ECFCs. This feature was not due to the downregulation of VEGFR2, the receptor isoform that mediates its pro-angiogenic effect also in ECFCs [52], and suggested that the signalling pathways downstream VEGFR2 could be silenced/suppressed in tumor-associated ECFCs. Intracellular Ca^2+^ oscillations represent the main mode whereby growth factors stimulate angiogenesis in both mature endothelial cells [38, 39] and their more immature precursors [28, 29]. An increase in [Ca^2+^]_i_ represents a central hub in the sophisticated network of interconnected signalling pathways that are recruited following VEGFR2 stimulation. For instance, intracellular Ca^2+^ oscillations deliver the most effective signal to engage the Ca^2+^-dependent transcription factors, NF-κB and nuclear factor of activated T cells (NFAT), which play a key role in VEGF-induced ECFC proliferation [28, 53], extracellular signal-regulated kinase (ERK), and phosphatidyl inositol 3-kinase (PI3K)/Akt/protein kinase B [54], following VEGF stimulation. Our Ca^2+^ imaging recordings revealed that VEGF-induced intracellular Ca^2+^ oscillations were dramatically down-regulated in BC-ECFCs as compared to healthy cells. This observation is fully consistent with the results obtained from other types of tumor-associated ECFCs. Accordingly, VEGF failed to induce detectable Ca^2+^ spikes in RCC- and IH-ECFCs [24, 25], although VEGFR2 was normally expressed in these cells. Similarly, VEGF-induced Ca^2+^ oscillations were rather weak in ECFCs isolated from individuals affected from PMF [26], a chronic myeloproliferative neoplasm that is characterized by the development of a robust vascular network in both the bone marrow and spleen. Interestingly, VEGF failed to induce proliferation and tube formation also in these cells, a finding that has been invoked to explain the failure of anti-VEGF in this disease [13, 26, 34]. We, therefore, suggest that the weaker Ca^2+^ burst induced by VEGF in BC-ECFCs and PMF-ECFCs as compared to N-ECFCs does not reach the threshold of activation of endothelial Ca^2+^-dependent pro-angiogenic transcription factors, such as NF-κB and NFAT.

The down-regulation of VEGF-induced intracellular Ca^2+^ oscillations could depend on the recruitment of signalling components other than those at work in N-ECFCs [26] or on the remodelling of the Ca^2+^ toolkit [24, 25, 35]. However, the following pieces of evidence confirmed that the PLCγ/InsP_3_/SOCE signalling pathway was engaged by VEGF also in BC-ECFCs. First, the Ca^2+^ signal arose in the absence of extracellular Ca^2+^, which indicated that the Ca^2+^ response was driven by intracellular Ca^2+^ mobilization rather than Ca^2+^ entry, as described in PMF-ECFCs [26]. Second, the pharmacological blockade of PLCγ with U73122 or of InsP_3_Rs with 2-APB abrogated the onset of the Ca^2+^ spikes. Third, the pharmacological blockade of SOCE with BTP2 mimicked the effect of 0Ca^2+^ by curtailing the duration of the Ca^2+^ train without preventing its onset. Unlike 0Ca^2+^ conditions, however, BTP2 did not delay the onset of the 1^st^ Ca^2+^ spike. This apparent discrepancy could be explained by anticipating that BTP does not fully abrogate SOCE in BC-ECFCs (see Figure 7). We hypothesize that SOCE represents the source of Ca^2+^ necessary to sensitize InsP_3_Rs to PLCγ-derived InsP_3_, by acting either on the luminal or the cytosolic side [55, 56], thereby regulating the latency of the 1^st^ Ca^2+^ spike. If BTP2 does not fully abrogate SOCE, then some extremely localized Ca^2+^ influx is predict to occur in proximity of InsP_3_Rs and maintain the latency of the signal unaltered. Obviously, no Ca^2+^ entry occurs in the absence of external Ca^2+^, which could lead to a significant delay in the onset of the oscillations.

Based on the evidences illustrated above, the most likely interpretation to account for the attenuation of the pro-angiogenic Ca^2+^ oscillations was the remodelling of the Ca^2+^ toolkit in BC-ECFCs. This phenomenon has recently been proposed to underlie the resistance to chemotherapy and radiation therapy in both tumor cells [57, 58] and tumor-associated ECFCs [23, 34]. For instance, the reduction in ER Ca^2+^ concentration ([Ca^2+^]_ER_) and the hypo-expression of InsP_3_Rs prevent VEGF from triggering robust Ca^2+^ spikes in RCC-ECFCs [24, 35]. Our Ca^2+^ imaging recordings revealed that the ER Ca^2+^ pool was reduced also in BC-ECFCs. Accordingly, CPA-induced intracellular Ca^2+^ release was significantly dampened as compared to healthy cells. CPA, as well as its structurally unrelated analogue, thapsigargin, unmasks the physiological Ca^2+^ leakage through ER membrane by inhibiting SERCA-mediated Ca^2+^ sequestration, thereby leading to the rapid depletion of the ER Ca^2+^ pool. Previously, we exploited this strategy to show that ER Ca^2+^ levels were decreased in RCC-, IH-, and PMF-ECFCs [24, 25, 47]. Notably, the chronic underfilling of ER in tumor-associated ECFCs was confirmed by directly measuring [Ca^2+^]_ER_ through recombinant ER-targeted aequorin [35, 59]. This observation was further supported by the finding that InsP_3_-dependent Ca^2+^ release, which was monitored by challenging the cells with the InsP_3_-synthesizing autacoid ATP [24, 44], was also smaller in BC-ECFCs, while InsP_3_Rs were normally expressed. On the other hand, there was no difference in the amplitude and molecular composition of SOCE between N- and BC-ECFCs. Consistently, there was no difference in the expression profile of Orai1 and TRPC1, which provide the Ca^2+^-permeable routes on the plasma membrane gated following ER depletion, between N- and BC-ECFCs. The overexpression of Stim1, which functions as the sensor of [Ca^2+^]_ER_ and activates SOCs, was not sufficient to enhance SOCE in the latter, as previously shown in human salivary gland cells [60]. This feature confirms that Orai1 and TRPC1 represent the two limiting structural components of the SOCE machinery and that a correct stoichiometric expression of Stim1, Orai1 and TRPC1 is necessary for full SOCE activation in ECFCs [37, 61]. Moreover, SOCE was inhibited by BTP2 and 10 μM La^3+^, which block SOCs contributed by Orai1 and TRPC1 in a growing number of cell types [36, 62–65], including tumor-associated ECFCs [24, 25]. Unlike IH-ECFCs [25], pre-incubating the cells with either BTP2 or La^3+^ did not cause the depletion of the InsP_3_-sensitive ER Ca^2+^ pool. This result suggests that SOCE is not, or just minimally, activated under resting conditions and requires agonist-induced ER depletion to arise. Altogether, these observations strongly suggest that the down-regulation of VEGF-induced Ca^2+^ oscillations is due to the reduction in [Ca^2+^]_ER_, which prevents InsP_3_ from triggering the dynamic interaction between InsP_3_-dependent Ca^2+^ release and SOCE which reliably engages the Ca^2+^-dependent pro-angiogenic genetic program in N-ECFCs. The drop in ER Ca^2+^ levels could be due to the up-regulation of TMTC1 recently reported in BC-ECFCs [22]. TMTC1 is a novel ER-resident tetratricopeptide repeat-containing adapter protein that binds to SERCA2B to curb its activity [66]. It has been shown that over-expression of TMTC1 in HEK293T caused a strong reduction in acetylcholine- and ionomycin-induced intracellular Ca^2+^ mobilization [66]. Although this hypothesis remains to be experimentally probed, we speculate that the increase in TMTC1 levels leads to the chronic Ca^2+^ underfilling of ER in BC-ECFCs. Likewise, future work will have to address whether lysosomal Ca^2+^ signalling contributes to VEGF-induced intracellular Ca^2+^ oscillations and is dysregulated in BC-ECFCs. Accordingly, nicotinic acid adenine dinucleotide phosphate (NAADP) has been recently shown to underpin VEGF-induced endothelial Ca^2+^ signals and neo-angiogenesis in melanoma [67]. An NAADP-sensitive lysosomal Ca^2+^ store is also present in N-ECFCs [30, 68], although it is seemingly down-regulated in BC-ECFCs (unpublished observations from our group).

As widely discussed elsewhere [13, 23], ECFC insensitivity to VEGF could contribute to the resistance to anti-VEGF therapies observed in cancer patients. Accordingly, ECFCs resident within the vascular “stem cell niches” provide the building blocks for neovessel formation in growing tumors. Additionally, ECFCs paracrinally may boost angiogenesis by releasing a myriad of growth factors and cytokines that stimulate endothelial cells to undergo angiogenesis [13, 16–21, 69, 70]. Limited evidence has been provided to show that human TECs require VEGF for proliferation, survival and migration [20, 71–73], while only one study revealed VEGF-induced Ca^2+^ signals in B-TECs [72]. In the clinical practice, anti-VEGF inhibitors are administered as adjuvant for standard chemotherapy or radiation therapy when tumor vasculature has already been established. At this stage, ECFCs have already been diluted/replaced by endothelial cells sprouting from neighbouring capillaries and B-TEC mainly derive from VEGF-sensitive cancer stem cells or adjoining sprouting capillaries [12, 74–76]. It turns out that tumor blood vessels, which are mainly lined by VEGF-sensitive B-TECs, regress in the presence of anti-angiogenic inhibitors. We hypothesize that the consequent dismantling of tumor vasculature exacerbates the hypoxic conditions of tumor microenvironment, thereby boosting the activation of hypoxia-inducible factors (HIFs) and inducing a second wave of ECFC mobilization [23]. Consequently, circulating ECFCs will be again recruited to the tumor site, in which they will be able to proliferate and re-establish the vascular network in spite of the presence of anti-VEGF drugs as they are not sensitive to VEGF [13, 23]. Although this scenario remains speculative and does not rule out the contribution of other mechanisms to the development of acquired refractoriness, including VEGFR2 downregulation in B-TECs [77], it could explain the limited increase in OS and PFS observed in BC patients treated with anti-angiogenic inhibitors. Unfortunately, no study has hitherto assessed the impact of anti-VEGF drugs on ECFC frequency either in BC or in any other tumor type. Of note, earlier studies showed that the systemic administration of bevacizumab caused an increase in the frequency of CD45^dim^, CD133^+^, VEGFR2^+^ EPCs in BC patients not responding to the therapy, while a reduction could not always be observed in those who did not show any change in disease progression [78]. Likewise, there was no significant relationship between the frequency of CD45^–^, CD133^+^/CD34^+^_EPCs and the therapeutic outcome of bevacizumab in BC patients enrolled in another study [79].

If VEGF does not stimulate BC-ECFC proliferation and tube formation, VEGFR2 cannot serve as a suitable target to prevent or interfere with BC vascularization. Nevertheless, the finding that the pharmacological blockade of SOCE with either BTP2 or 10 μM La^3+^ suppresses BC-ECFC growth and *in vitro* tubulogenesis provides further hints at SOCE as a promising candidate to develop alternative treatments to treat BC [36, 80]. Several studies showed that SOCE drives proliferation and migration also in several BC cell lines [43, 81, 82]. Therefore, SOCE stands out as a very attractive target to simultaneously halt BC cell growth and prevent ECFC activation at the tumor site [23, 33]. It would be interesting to assess whether SOCE controls proliferation (or migration) also in B-TECs, in which only store-independent Ca^2+^ pathways have hitherto been described [72, 83]. The rationale to include SOCE among the most promising molecular anti-cancer targets has been provided by pre-clinical and clinical studies conducted on CAI. CAI is a non-specific synthetic blocker of various types of Ca^2+^ channels, including InsP_3_Rs, TRP channels, SOCs, and the mitochondrial Ca^2+^ uniporter [33, 84]. CAI suppresses a multitude of Ca^2+^-dependent signalling pathways and has been validated as an effective anti-tumor agent due to its ability to inhibit angiogenesis as well as tumor development, migration and metastasis [33]. Preliminary studies revealed that CAI also blocked proliferation and/or migration in several human BC cell lines and in B-TECs [85]. Previous work from our group confirmed that CAI prevented N- and RCC-ECFC proliferation and *in vitro* tubulogenesis by interfering with both ER-dependent Ca^2+^ release and SOCE activation [24]. The present results extend these observations by showing that CAI interferes with intracellular Ca^2+^ signalling and inhibits both growth and tube formation also in BC-ECFCs. Of note, CAI represents the sole Ca^2+^ antagonist currently under investigation as an orally administered tumoristatic and anti-angiogenic agent in clinical phase I-III trials of several solid cancers (http://clinicaltrials.gov/). Future work will have to assess whether CAI is capable of halting BC vascularization and causing tumor shrinkage also in preclinical models.

In conclusion, we provided the first clear-cut evidence that VEGF does not stimulate angiogenesis in ECFCs isolated from peripheral blood of metastatic BC patients. Several authors proposed that the acquired resistance to anti-VEGF therapy could result from the recruitment of vascular progenitor cells and myeloid cells from their bone marrow/vascular niches, which may obviate the necessity of VEGF signalling [7, 8, 11, 23, 86]. Therefore, our data further corroborate the notion that tumor-associated ECFCs are insensitive to VEGF and lend the first strong cellular and molecular support to the notion that VEGFR2 is unlikely to be the most suitable target to halt vascularization and prevent tumor relapse in BC patients. VEGF fails to stimulate proliferation in BC-ECFCs due to the down-regulation of the intracellular Ca^2+^ spikes arising in normal cells following VEGFR-2 activation. The dramatic reduction in ER Ca^2+^ content is a key determinant to prevent VEGF from triggering the periodic InsP_3_-depedent Ca^2+^ release that underlies the Ca^2+^ train. This feature further suggests that the remodelling of the Ca^2+^ toolkit stands out as a crucial player in the development of the refractoriness to anti-cancer treatments [23, 57, 58]. Targeting the intracellular Ca^2+^ machinery could represent an alternative, yet promising strategy to overcome the resistance to anti-angiogenic therapies and prevent tumor vascularization. In this view, SOCE is emerging as an attractive candidate to design innovative approaches to treat BC patients as it controls proliferation in both BC cells and BC-ECFCs, as shown by the present investigation.

## MATERIALS AND METHODS

### Isolation and cultivation of ECFCs

Blood samples (40 mL) were obtained from patients who were out of ongoing cytoreductive therapy at the moment of blood sampling for ECFC isolation and from healthy donors. The ECFC samples used in the present investigation belong to the stocks recently described and characterized in [22]. To isolate ECFCs, mononuclear cells (MNCs) were separated from peripheral blood by density gradient centrifugation on lymphocyte separation medium for 30 min at 400g and washed twice in endothelial basal medium-2 (EBM-2) with 2% foetal bovine serum (FBS). A median of 36 × 10^6^ MNCs (range 18-66) were plated on collagen-coated culture dishes (BD Biosciences) in the presence of the endothelial cell growth medium EGM-2 MV Bullet Kit (Lonza) containing EBM-2, 5% FBS, recombinant human (rh) EGF, rhVEGF, rhFGF-B, rhIGF-1, ascorbic acid and heparin, and maintained at 37°C in 5% CO_2_ and humidified atmosphere. Discard of non-adherent cells was performed after 2 days; thereafter medium was changed three times a week. The outgrowth of endothelial colonies from adherent MNCs was characterized by the formation of a cluster of cobblestone-appearing cells, resembling endothelial cells. That ECFCs-derived colonies belonged to endothelial lineage was confirmed as described in [22, 87]. In more detail, ECFCs-derived colonies were stained with anti-CD31, anti-CD105, anti-CD144, anti-CD146, anti-von Willebrand factor (vWF), anti-CD45, and anti-CD14 monoclonal antibodies and by assessment of capillary-like network formation in an *in vitro* Matrigel assay. For our experiments, we have mainly used endothelial cells obtained from early passage ECFCs (P1-3, which roughly encompasses a 15-18 day period) with the purpose to avoid (or maximally reduce) any potential bias due to possible cell differentiation. As already show in [24], the immunophenotype of both normal and tumor ECFCs does not change at different passages in culture. We also tested whether functional differences occurred when early (P2) and late (P6) passage-ECFCs were used by testing the *in vitro* capacity of capillary network formation in a Matrigel assay and found no differences between early and late passage ECFC-derived cells (data not shown).

### Solutions

Physiological salt solution (PSS) had the following composition (in mM): 150 NaCl, 6 KCl, 1.5 CaCl_2_, 1 MgCl_2_, 10 Glucose, 10 Hepes. In Ca^2+^-free solution (0Ca^2+^), Ca^2+^ was substituted with 2 mM NaCl, and 0.5 mM EGTA was added. Solutions were titrated to pH 7.4 with NaOH. The osmolality of PSS as measured with an osmometer (Wescor 5500, Logan, UT) was 338 mmol/kg.

### Electron microscopy

ECFCs were fixed with 2% paraformaldehyde and 2.5% glutaraldehyde (Sigma–Aldrich) in 0.1 M sodium phosphate buffer, pH 7.4, as described in [35]. After fixation, cells were postfixed in 2% osmium (OsO4) for 1 h at room temperature, dehydrated, and embedded in LR White (Sigma–Aldrich). Polymerization was performed for 24 h at 60°C. Ultrathin sections were cut with a Reichert (Depew, New York) OM-U3 ultramicrotome. The sections were stained with uranyl acetate and lead citrate (Sigma–Aldrich), examined and photographed at 3000×, 7.000×, or 20.000× magnifications on a Zeiss (Jena, Germany) EM900 (80 kV, objective diaphragm 30 μm) electron microscope.

### Flow cytometry

Early passage ECFCs were isolated by trypsinization and resuspended in EBM2 medium (Lonza, Basel CH) supplemented with 10% FBS, as described in [88]. ECFC-derived cells (1×10^5^ cells) were incubated with 5 μl of phycoerythrin (PE)-conjugated isotype control (IgG1) (BD Biosciences, San Josè, CA, USA) or 5 μl of PE-conjugated anti- VEGFR-2 antibody (R&D Systems, Inc Minneapolis, MN, USA) for 30 minutes at 4°C. ECFCs were than washed and resuspended in PBS with 1% FBS. Cells were aquired by a flow cytometer (Navios™; Beckman Coulter, Inc, Brea, CA) and analyzed by Kaluza^®^ flow analysis software (Beckman Coulter). The number of dead/apoptotic cells was negligible, therefore the analysis was performed excluding cellular debris in a side scatter/forward scatter dot plot.

### [Ca^2+^]_i_ measurements and statistical analysis of Ca^2+^ signals

ECFCs were loaded with 4 μM fura-2 acetoxymethyl ester (fura-2/AM; 1 mM stock in dimethyl sulfoxide) in PSS for 1 hour at room temperature. After washing in PSS, the coverslip was fixed to the bottom of a Petri dish and the cells observed by an upright epifluorescence Axiolab microscope (Carl Zeiss, Oberkochen, Germany), usually equipped with a Zeiss ×40 Achroplan objective (water-immersion, 2.0 mm working distance, 0.9 numerical aperture). ECFCs were excited alternately at 340 and 380 nm, and the emitted light was detected at 510 nm. A first neutral density filter (1 or 0.3 optical density) reduced the overall intensity of the excitation light and a second neutral density filter (optical density=0.3) was coupled to the 380 nm filter to approach the intensity of the 340 nm light. A round diaphragm was used to increase the contrast. The excitation filters were mounted on a filter wheel (Lambda 10, Sutter Instrument, Novato, CA, USA). Custom software, working in the LINUX environment, was used to drive the camera (Extended-ISIS Camera, Photonic Science, Millham, UK) and the filter wheel, and to measure and plot on-line the fluorescence from 10 up to100 rectangular “regions of interest” (ROI). Each ROI was identified by a number. Since cell borders were not clearly identifiable, a ROI may not include the whole cell or may include part of an adjacent cell. Adjacent ROIs never superimposed. [Ca^2+^]_i_ was monitored by measuring, for each ROI, the ratio of the mean fluorescence emitted at 510 nm when exciting alternatively at 340 and 380 nm (shortly termed “ratio”). An increase in [Ca^2+^]_i_ causes an increase in the ratio [87]. Ratio measurements were performed and plotted on-line every 3 s. The experiments were performed at room temperature (22°C).

All the data have been collected ECFCs isolated from three different healthy donors. VEGF-induced intracellular Ca^2+^ oscillations were analysed by evaluating: 1) the latency before the onset of the pacemaker potential leading to the first regenerative Ca^2+^ spike; 2) the amplitude of the first Ca^2+^ spike (which was calculated as the difference as the ratio at the peak and the mean ratio of 1 min baseline before the slow pacemaker potential); 3) the number of Ca^2+^ spikes during 1 hour recording. The amplitude of the peak Ca^2+^ response to CPA and ATP was measured as the difference between the ratio at the peak (either of intracellular Ca^2+^ mobilization in 0Ca^2+^ or of Ca^2+^ entry occurring upon Ca^2+^ restoration to the bath) and the mean ratio of 1 min baseline before the peak. Pooled data are given as mean±SE and statistical significance (p<0.05) was evaluated by the Student's *t* test for unpaired observations.

### Wavelet analysis of Ca^2+^ signals

To give a quantitative evaluation of the differences in Ca^2+^ oscillatory activity between N- and BC-ECFCs after VEGF administration, an approach based on wavelet analysis was employed, using KYM 0.5 software (http://sourceforge.net/projects/kym/) as presented in [26, 39, 40]. Briefly, the wavelet transform W(*a,b*) = |*a*|^-1/2^ ∫ ψ^*^[(*t – b*)/*a*] *f*(*t*) d*t* was computed for each calcium trace using Morlet function ψ(*t*) ≈ π^-1/4^ exp(–0.5 *t*^2^) exp(*ist*) as mother wavelet, then, after having renamed *b* as *t* and having done the substitution *ν* = *s*/(*2πa*), the activity index J(*t*) ≈ ∫ |W(*t,ν*)|^2^ *ν* d*ν* was calculated according to equations 3,4,5 and 9 of [40]. Notably, weighting wavelet transform modulus by the frequency, J keeps into account both the amplitudes and the values of the frequency components making up the oscillatory signal. Activity index has been evaluated for each calcium trace as a function of time, then its time-averaged value <J> has been calculated for each cell thus allowing to statistically compare the two <J> mean values representative of the two group of interest (N- and BC-ECFCs).

### RNA isolation and qRT-PCR

Total RNA was extracted from N- and BC-ECFCs by using the QIAzol Lysis Reagent (QIAGEN, Italy). Single cDNA was synthesized from RNA (1 μg) using random hexamers and M-MLV Reverse Transcriptase (Invitrogen S.R.L., Italy). Reverse transcription was always performed in the presence or absence (negative control) of the reverse transcriptase enzyme. qRT-PCR was performed in triplicate using 1 μg cDNA and specific primers (intron-spanning primers) for Stim1-2, Orai1-3, TRPC1-7, and InsP_3_Rs1-3, as previously described [24, 47] (Supplementary Tables 1-2). Briefly, GoTaq qPCR Mastermix (Promega, Italy) was used according to the manufacturer instruction and qRT-PCR performed using Rotor Gene 6000 (Corbett, Concorde, NSW, Australia). The conditions were as follows: initial denaturation at 95°C for 5 min; 40 cycles of denaturation at 95°C for 30 sec; annealing at 58°C for 30 sec, and elongation at 72°C for 40 sec. The qRT-PCR reactions were normalized using β-actin as housekeeping gene. Melting curves were generated to detect the melting temperatures of specific products immediately after the PCR run. The triplicate threshold cycles (Ct) values for each sample were averaged resulting in mean Ct values for both the gene of interest and the housekeeping gene β-actin. The gene Ct values were then normalized to the housekeeping gene by taking the difference: ΔCt = Ct[gene] - Ct[β-actin], with high ΔCt values reflecting low mRNA expression levels. The sequences of the bands were checked by using the Big dye terminator cycle sequencing kit (Applied Biosystem, PE, USA). PCR products were also separated with agarose gel electrophoresis, stained with ethidium bromide, and acquired with the Image Master VDS (Amersham Biosciences Europe, Italy). The molecular weight of the PCR products was compared to the DNA molecular weight marker VIII (Roche Molecular Biochemicals, Italy).

### Sample preparation and immunoblotting

ECFCs were homogenized by using a Dounce homogenizer in a solution containing: 250 mM Sucrose, 1 mM EDTA, 10 mM Tris-HCl, pH 7.6, 0.1 mg/mL PMSF, 100 mM β-mercaptoethanol and Protease Inhibitor Cocktail (P8340, Sigma, USA). The homogenates were solubilized in LaemmLi buffer (Dragoni et al., 2014) and 30 μg proteins were separated on 10% SDS-polyacrilamide gel electrophoresis and transferred to the Hybond-P PVDF Membrane (GE Healthcare, Italy) by electroelution. After 1 h blocking with Tris buffered saline (TBS) containing 3% BSA and 0.1% Tween (blocking solution), the membranes were incubated for 3 h at room temperature with the following affinity purified antibodies diluted 1:200 in the TBS and 0.1% Tween: anti-Stim1 (sc-166840), anti-Orai1 (sc-68895), anti-TRPC1 (sc-133076), anti-TRPC3/6/7 (sc-15056), and anti-IP3R-I/II/III (sc-377518) from Santa Cruz Biotechnology, anti-Orai3 (HPA015022), anti-Stim2 (PRS4123) from Sigma-Aldrich (Italy), and anti β-actin rabbit antibody as control (Rockland Immunochemicals for Research, U.S.A.; code, 600-401-886). The membranes were washed and incubated for 1 h with peroxidase-conjugated mouse, rabbit or goat IgG (1:120000 in blocking solution), from Dakocytomation (P0260), Chemicon (AP132P), and Santa Cruz (sc-2354), respectively. The bands were detected with the ECL™ Select western blotting detection system (GE Healthcare Europe GmbH, Italy). Prestained molecular weight markers (SDS7B2, Sigma, Italy) were used to estimate the molecular weight of the bands. Control experiments were performed by using the antibody preadsorbed with a 20-fold molar excess of the immunizing peptide or by incubating the blots with non immune serum.

### Protein content

Protein contents of all the samples were determined by the Bradford's method using bovine serum albumin (BSA) as standard [24, 47].

### Chemicals

EBM and EGM-2 were purchased from Clonetics (Cell System, St. Katharinen, Germany). Fura-2/AM was obtained from Molecular Probes (Molecular Probes Europe BV, Leiden, The Netherlands). BTP2 was purchased from Calbiochem (La Jolla, CA, USA). All other chemicals were obtained from Sigma Chemical Co. (St. Louis, MO, USA).

### Proliferation assays

As described elsewhere [24, 87], growth kinetics were evaluated by plating a total of 1 × 10^5^ ECFCs-derived cells (first passage) in 30-mm collagen-treated dishes in the presence of EBM-2 and 5% FBS supplemented with 10 ng/mL VEGF and one of the either compounds: BAPTA (30 μM, 1 hour), BTP2 (20 μM, 30 min), La^3+^ (10 μM, 30 min), or CAI (10 μM, 20 min). Cultures were incubated at 37°C (in 5% CO2 and humidified atmosphere) and cell growth assessed every day until confluence was reached in control cultures. At this point, cells were recovered by trypsinization from all dishes and the cell number assessed by counting in a haemocytometer. Preliminary experiments showed no unspecific or toxic effect for each agent when used at these concentrations. Each assay was repeated in triplicate.

### *In vitro* tube formation assay

The tube formation assay was carried out as previously shown [26]. To evaluate the effect of VEGF, early passage (P2-P3) BC-ECFCs were detached by trypsinization and resuspended in EBM-2 supplemented with 2% FBS. ECFC-derived cells were plated at 1.5 × 10^4^ per well in Cultrex (Trevigen)-coated 96 well plates, in the presence of EBM-2 ± 10 ng/mL VEGF. Capillary network formation was assessed starting from 4 to 24 hours later. Three different sets of experiments, each performed in duplicate, were carried out. Experiments were repeated a minimum of three times and the vasculogenic response was measured by evaluating both dimensional and topological parameters. As illustrated in [26], we analyzed the length of endothelial tube-like structures (TLSs) and the number of the polygon structures established by TLSs, which are referred to as complex meshes and are indicative of endothelial cell migration. These analyses were performed by using the Angiogenesis Analyzer plugin of ImageJ (Gilles Carpentier, Faculte´ des Sciences et Technologie, Universite´ Paris Est, Creteil Val de Marne, France) [26]. To evaluate the effect of SOCE inhibitors, the same protocol was repeated by stimulating *in vitro* angiogenesis with the EGM-2 medium in the presence and absence of the following drugs: BAPTA (30 μM, 1 hour), BTP2 (20 μM, 30 min), or CAI (10 μM, 20 min).

## SUPPLEMENTARY MATERIALS FIGURES AND TABLES


