# Supplementary material for: VEGF-induced intracellular Ca2+ oscillations are down-regulated and do not stimulate angiogenesis in breast cancer-derived endothelial colony forming cells
Source: Oncotarget. 2017 Aug 14;8(56):95223–46. doi: 10.18632/oncotarget.20255 (PMC5707017; doi:10.18632/oncotarget.20255)
Supplement: Supplementary file 1 [file oncotarget-08-95223-s001.pdf]

VEGF-induced intracellular Ca^{2+} oscillations are down-regulated and do not stimulate angiogenesis in breast cancer-derived endothelial colony forming cells

SUPPLEMENTARY MATERIALS

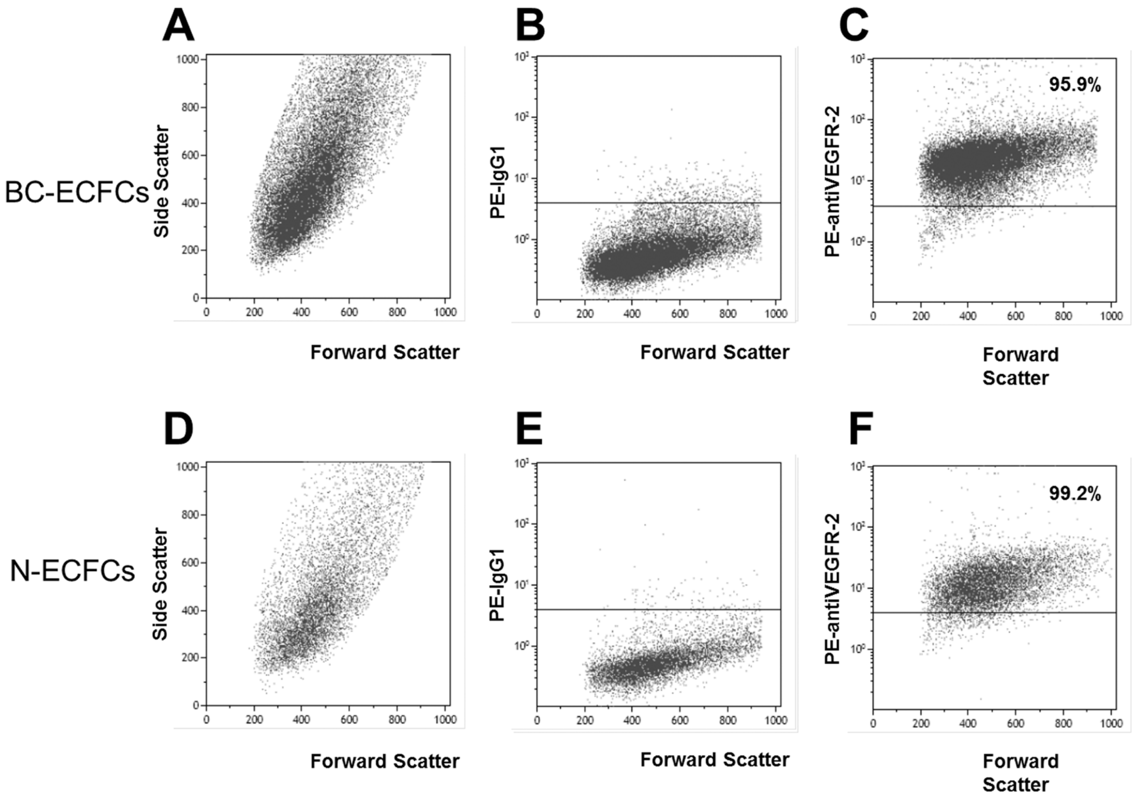

Supplementary Figure 1: Comparable expression of VEGFR-2 on normal and breast cancer-derived endothelial colony forming cells. Viable cultured ECFCs, as shown by the typical picture in the forward scatter/side scatter plots **(A** and **D)**, stained with the PE-IgG1 isotype control **(B** and **E)** and with the Pe-anti-VEGFR-2 antibody **(C** and **F)**.

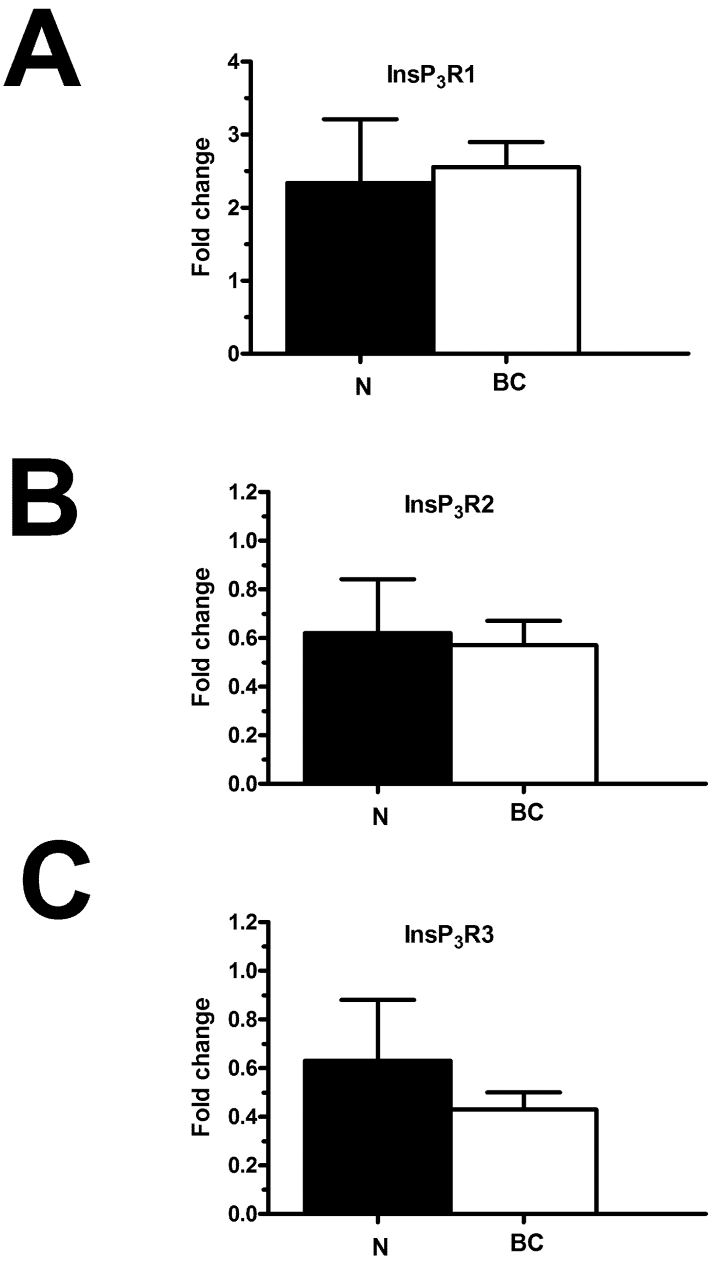

Supplementary Figure 2: InsP$_3$ receptors are normally expressed in breast cancer-derived endothelial colony forming cells. There is no difference in the expression of InsP$_3$R1 **(A)**, InsP$_3$R2 **(B)** and InsP$_3$R3 **(C)** between normal and breast cancer-derived ECFCs. mRNA levels were measured by RT-PCR relative to the β-actin internal standard (see Materials and Methods) and the values obtained were reported as ΔCt. Bars represent the mean±SE of at least 4 different experiments each from different RNA extracts. $^{*}P<0.05$ versus InsP$_3$R1 (1-way ANOVA followed by Newman–Keuls's Q test). The PCR products were of the expected size: InsP$_3$R1, 180 bp; InsP$_3$R2, 158 bp; InsP$_3$R3, 173 bp. The specific primers described in Supplementary Table 2 have been utilized to examine the expression levels of InsP$_3$R transcripts.

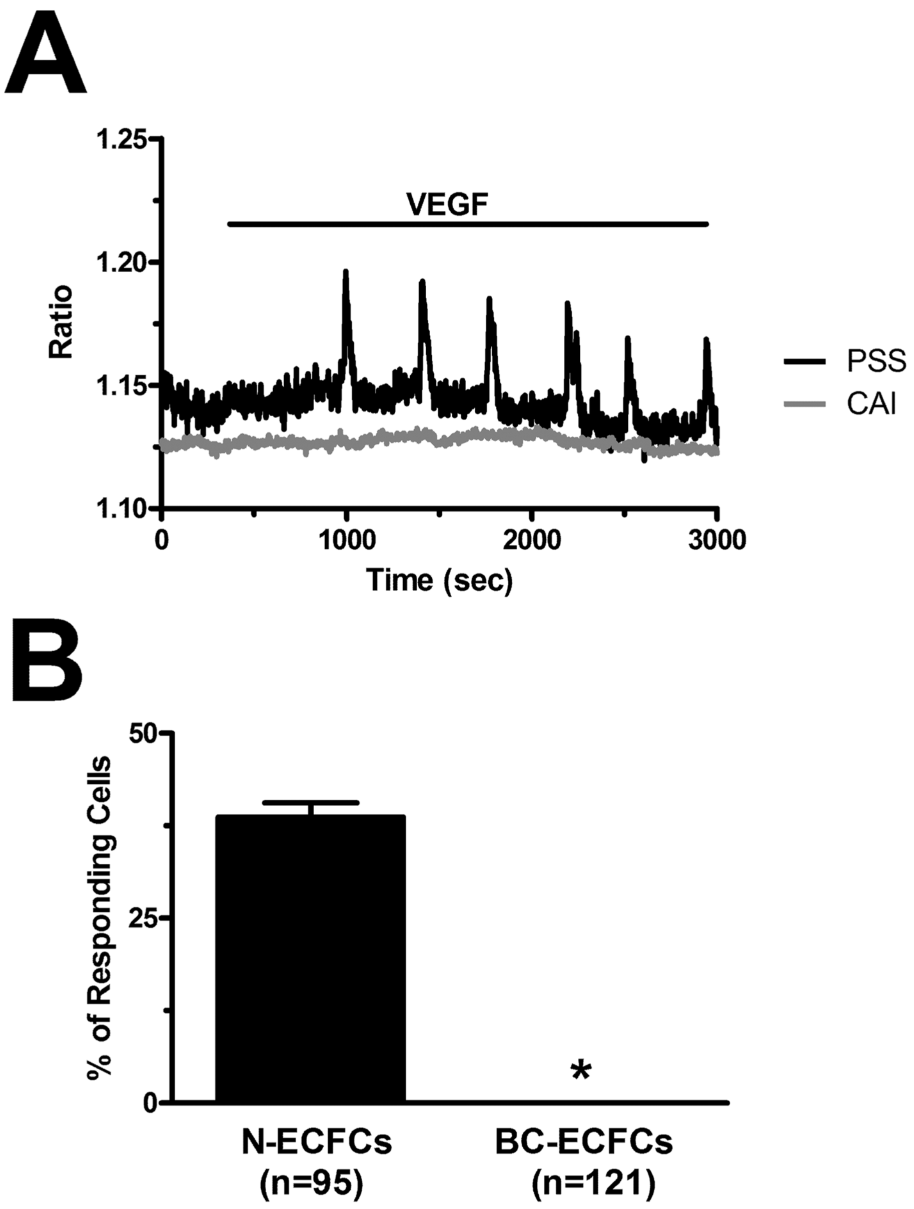

Supplementary Figure 3: CAI inhibits VEGF-induced intracellular Ca^{2+} oscillations in breast cancer-derived endothelial colony forming cells. **(A)**, CAI (10 µM, 20 min) prevents VEGF-induced intracellular Ca^{2+} spikes in BC-ECFCs. **(B)**, mean±SE of the percentage of VEGF-responding cells in the absence and the presence of CAI. The asterisk indicates $p<0.05$.

Supplementary Table 1: Primer sequences used for real time reverse transcription/polymerase chain reaction of $InsP_3$ receptors

Gene	Primer sequences		Size (bp)	Accession number
$InsP_3R1$	Forward	5’- TCAACAAACTGCACCACGCT -3’	180	ENSG00000150995
	Reverse	5’- CTCTCATGGCATTCTTCTCC -3’		
$InsP_3R2$	Forward	5’- ACCTTGGG GTTAGTGGATGA -3’	158	ENSG00000123104
	Reverse	5’- CCTTGTTTGGCTTGCTTTGC -3’		
$InsP_3R3$	Forward	5’- TGGCTTCATCAGCACTTTGG -3’	173	ENSG00000096433
	Reverse	5’- TGTCCTGCTTAGTCTGCTTG -3’		
β-actin		Hs_ACTB_1_SG, QuantiTect Primer Assay QT00095431, Qiagen	146	NM_001101

Supplementary Table 2: Primer sequences used for real time reverse transcription/polymerase chain reaction of TRPC1-7, Stim1-2 and Orai1-3

Gene	Primer sequences		Size (bp)	Accession number
TRPC1	Forward	5'-ATCCTACACTGGTGGCAGAA-3'	307	NM_003304.4
	Reverse	5'-AACAAAGCAAAGCAGGTGCC-3'		
TRPC3	Forward	5'-GGAGATCTGGAATCAGCAGA-3'	336	NM_001130698.1 variant 1
	Reverse	5'-AAGCAGACCCAGGAAGATGA-3'		NM_003305.2 variant 2
TRPC4	Forward	5'-ACCTGGGACCTCTGCAAATA-3'	300	NM_016179.2 variant alpha
	Reverse	5'-ACATGGTGGCACCAACAAAC-3'		NM_001135955.1 variant beta
				NM_001135956.1 variant gamma
				NM_001135957.1 variant delta
				NM_003306.1 variant epsilon
				NM_001135958.1 variant zeta
TRPC5	Forward	5'-GAGATGACCACAGTGAAGAG-3'	221	NM_012471.2
	Reverse	5'-AGACAGCATGGGAAACAGGA-3'		
TRPC6	Forward	5'-AGCTGTTCCAGGGCCATAAA-3'	341	NM_004621.5
	Reverse	5'-AAGGAGTTCATAGCGGAGAC-3'		
TRPC7	Forward	5'-CACTTGTGGAACCTGCTAGA-3'	387	NM_020389.1
	Reverse	5'-CATCCCAATCATGAAGGCCA-3'		
Orai1	Forward	5'-AGTTACTCCGAGGTGATGAG-3'	257	NM_032790.3
	Reverse	5'-ATGCAGGTGCTGATCATGAG-3'		
Orai2	Forward	5'-CCATAAGGGCATGGATTACC-3'	334	NM_001126340.1 variant 1
	Reverse	5'-CAGGTTGTGGATGTTGCTCA-3'		NM_032831.2 variant 2
Orai3	Forward	5'-CCAAGCTCAAAGCTTCCAGCC-3'	159	NM_152288.2
	Reverse	5'-CAAAGAGGTGCACAGCCACCA-3'		
Stim1	Forward	5'-CCTCAGTATGAGGAGACCTT-3'	347	NM_003156.3
	Reverse	5'-TCCTGAAGGTCATGCAGACT-3'		
Stim2	Forward	5'-AAACACAGCCATCTGCACAG-3'	186	NM_020860.2
	Reverse	5'-GGGAAGTGTCGTTCCTTTGA -3'		
β-actin		Hs_ACTB_1_SG, QuantiTect Primer Assay QT00095431, Qiagen	146	NM_001101
